# Task-irrelevant auditory metre shapes visuomotor sequential learning

**DOI:** 10.1007/s00426-022-01690-y

**Published:** 2022-06-12

**Authors:** Alexis Deighton MacIntyre, Hong Ying Josephine Lo, Ian Cross, Sophie Scott

**Affiliations:** 1 Institute of Cognitive Neuroscience, University College London, London, UK; 2 Centre for Music and Science, University of Cambridge, Cambridge, UK; 3 MRC Cognition and Brain Sciences Unit, University of Cambridge, Cambridge, UK

## Abstract

The ability to learn and reproduce sequences is fundamental to every-day life, and deficits in sequential learning are associated with developmental disorders such as specific language impairment. Individual differences in sequential learning are usually investigated using the serial reaction time task (SRTT), wherein a participant responds to a series of regularly timed, seemingly random visual cues that in fact follow a repeating deterministic structure. Although manipulating inter-cue interval timing has been shown to adversely affect sequential learning, the role of metre (the patterning of salience across time) remains unexplored within the regularly timed, visual SRTT. The current experiment consists of an SRTT adapted to include task-irrelevant auditory rhythms conferring a sense of metre. We predicted that (1) participants’ (*n* = 41) reaction times would reflect the auditory metric structure; (2) that disrupting the correspondence between the learned visual sequence and auditory metre would impede performance; and (3) that individual differences in sensitivity to rhythm would predict the magnitude of these effects. Altering the relationship via a phase shift between the trained visual sequence and auditory metre slowed reaction times. Sensitivity to rhythm was predictive of reaction times over all. In an exploratory analysis, we, moreover, found that approximately half of participants made systematically different responses to visual cues on the basis of the cues’ position within the auditory metre. We demonstrate the influence of auditory temporal structures on visuomotor sequential learning in a widely used task where metre and timing are rarely considered. The current results indicate sensitivity to metre as a possible latent factor underpinning individual differences in SRTT performance.

**Supplementary Information:**

The online version contains supplementary material available at 10.1007/s00426-022-01690-y.

## Introduction

**Fig. 1:**
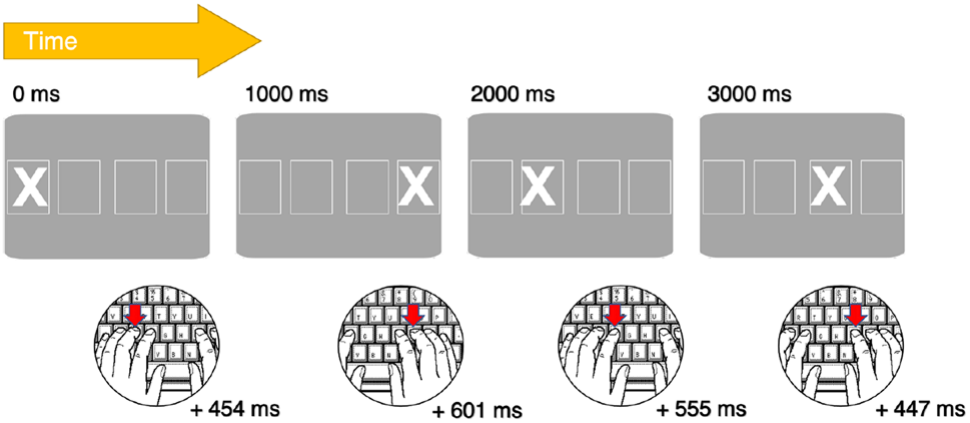
The Serial Reaction Time Task (Nissen & Bullemer, 1987) entails a participant making repeated motor responses to a series of visual cues, which may appear to be random, but are in fact deterministic or probabilistic in structure. Over many trials, the participant’s reaction times increase in speed, until the trained series changes to a new sequence of cues, at which point reaction times slow again. This slowing in response to a new sequence is interpreted as an indirect measure of sequential learning

Sequences, the serial ordering of events and actions, structure many of the ways in which we interact with our environment and each other. From humans to songbirds (Sainburg et al., 2019) to whales (Cholewiak et al., 2013), sequences are common to animals with rich social lives and aptitude for flexible cultural transmission. Sequences by their nature unfold across time, and data from electroencephalography (EEG) suggest that, when preparing to execute an action sequence, sequential timing is as integral to motor planning as the content of the sequence itself (Bortoletto et al., 2011). The temporal organisation of successive events or actions is referred to as rhythm, which is vividly expressed in music, dance, and poetry; however, more banal forms of rhythm also permeate throughout every-day life, for example, in the form of stereotyped timing that emerges when chopping vegetables or typing on a keyboard. Despite the centrality of rhythm to sequences, timing is infrequently considered in the context of sequential learning. The serial reaction time task (SRTT), for example, is arguably the most popular and comprehensively studied paradigm for this domain (Cleeremans et al., 1998; Destrebecqz & Cleeremans, 2001; Schwarb & Schumacher, 2012; Shanks, 2005), but it is primarily interpreted as a spatial-ordinal task. The SRTT entails a participant making repeated motor responses to a series of visual cues, which are usually presented at regular intervals (Nissen & Bullemer, 1987) (Fig. 1). If, however, the inter-cue intervals randomly vary, performance is poor, suggesting that unpredictable sequential rhythms may impede learning (Stadler, 1995). However, there are other temporal aspects of the task that are largely unexplored, such as *metre*.

**Fig. 2:**
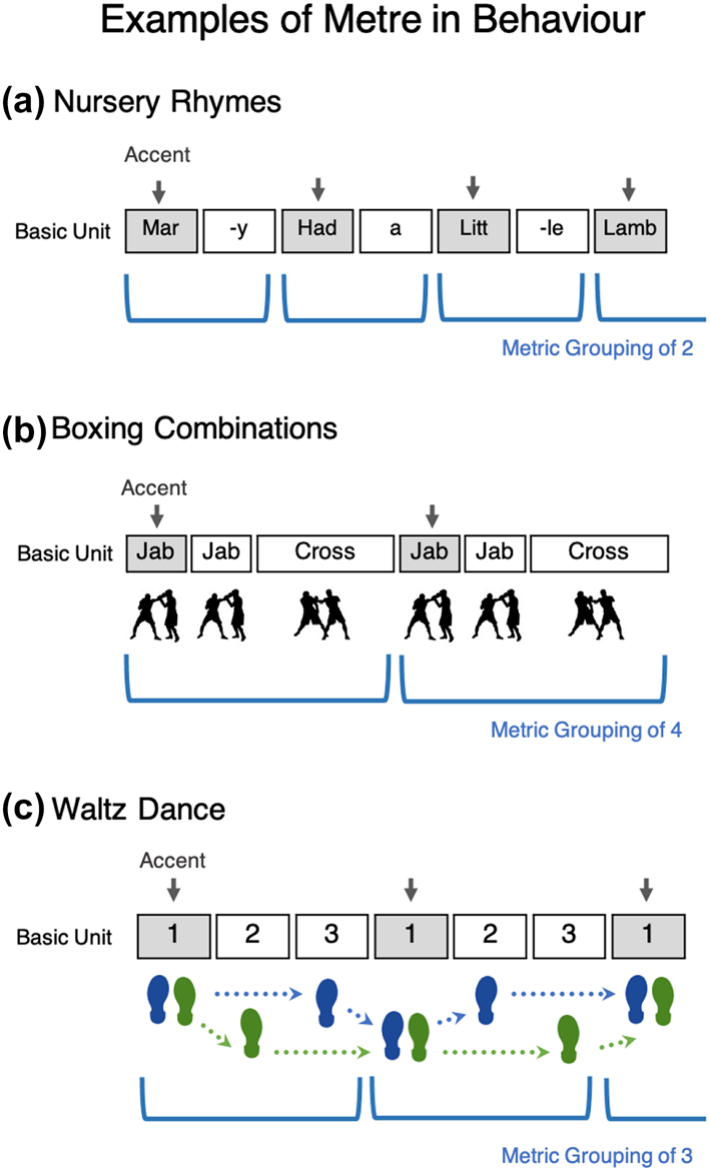
Metre is a central component of rhythm that functions as a structure or grid underlying events in time. Events within a metre that are perceived or enacted more strongly are said to be *accented*. Shown here are a few common examples of metre. **a** Depicts an English nursery rhyme, which is structured according to a binary pattern of accented (or stressed) and unaccented syllables, resulting in a grouping of two. In (**b**), boxers practice combinations of hits according to a fast–fast–slow metrical pattern, amounting to a grouping of four counts in total. Finally, **c** consists of a simple waltz dance step pattern, where an accented (or strong) beat is followed by two unaccented beats, invoking a metric grouping of three

Metre is a key component of human rhythm perception and production (Thaut et al., 2014), and can be described as a structure or grid underlying rhythmic events (every-day examples of metre are shown in Fig. 2). In Western popular music, for example, there is often a sense of pulse underlying the music, along which one might clap or nod their head. This pulse is known as the beat, and it is typical for the first of every four beats to be emphasised, resulting in the percept of a metre of four (i.e., a recurring grouping of four beats). In other musical cultures, such as in the Balkans or West Africa, metric groupings of five, six, nine, or other integers can also be common. Metre is also important in many forms of poetry. For example, the trochee metre is formed by the binary pattern of stressed and unstressed syllables. English nursery rhymes nearly always conform to a metre, but even when speaking prosaically, adults intuitively make their speech more metrical when addressing small children (Leong et al., 2017). Other human movement sequences can also be described within the framework of metre; for instance, the coordination of full body movements, such as running, tends to follow metric patterns across effectors [i.e., ratios of 1:2, 1:3, and 1:4, Bramble and Carrier (1983), MacPherson et al. (2009), Beek et al. (2003)].

Although conscious awareness of metre tends to be limited to certain contexts, such as the memorisation of dance steps, sensitivity to metric structure could also help us to parse dynamic processes throughout every-day life, thereby facilitating learning (deCastro Arrazola & Kirby, 2019; Van Peer, 1990) and influencing our motor planning (Palmer & Pfordresher, 2003). One reason may be that accents, defined as positions (relative to the metre) with greater salience, focus attention and enhance the readiness to act (Jones, 1976; Large & Jones, 1999). Hence, should a perceived accent coincide with relevant events or information, there could be a behavioural advantage associated with this opportunistic sensitivity to metre in temporal patterns in the environment. Indeed, evidence suggests that we sometimes even imagine or involuntarily impose metric patterns where none objectively exist, such as in the common tendency to perceive a “tick-tock” pattern within a clock’s objectively identical ticking sounds (Vlek et al., 2011).

We hypothesise that if a metre were to co-occur with an ongoing sequence to be learned, it is possible that the metric structure could interact with sequential elements, resulting in some representation of the metre in the response to, or reproduction of, that sequence. Specifically, heightened expectancy at the moment of metrical accent could temporarily boost performance, resulting in faster responses to expected visual cues. Over time, these same cues may also be learned more quickly, as evidenced by steeper declines in reaction times across many sequential presentations. Moreover, if auditory metre is integrated with visual sequential content, perturbing the relationship between the metre and sequence could also negatively impact the speed of RT. The current experiment directly addresses these possibilities by introducing two contrasting, task-irrelevant auditory metres to the SRTT paradigm in a preliminary exploration of metre in sequential learning.

### The serial reaction time task

In its most basic form, the SRTT consists of a series of location-based visual cues, normally represented as symbols or blocks on a computer screen. The participant’s task is to simply respond to each prime as quickly as possible by pressing its unique response key. Although no mention of any pattern is made and participants are not instructed to learn a sequence explicitly, the order in which the cues appear is actually a deterministic series that repeats throughout the training blocks. After numerous repetitions, participants’ reaction times (RT) gradually decrease. To dissociate sequence learning from more general motor practice effects, a test block containing an unfamiliar order of cues is presented in later stages of the experiment. The abrupt increase in RT that usually follows the introduction of the novel sequence is taken to index a participant’s implicit procedural knowledge of the learned sequence.

In addition to content learning, the SRTT can also be thought to have a temporal component. For example, the presentation of a new cue is usually timed relative to the previous stimulus (response to stimulus interval; RSI). In this case, the participant’s actions trigger the upcoming cue, but a fixed stimulus rate or inter-stimulus interval (ISI) can also be implemented (Brandon et al., 2012; Du & Clark, 2017; Honda et al., 1998; Moisello et al., 2009). In the original study by Nissen and Bullemer (1987), a 500-ms RSI was used. Later work established that the length of RSI can affect learning outcomes in different ways (Miyawaki, 2006). For example, performance may be enhanced by a short RSI (250 ms) in comparison to no RSI at all (Destrebecqz & Cleeremans, 2001), but if the RSI is too long (1500 ms), learning can then also be reduced (Frensch & Miner, 1994).

Typically, RSI are held constant, but it was found that randomly inserting a 2-s pause into otherwise temporally regular RSI slows RT considerably (Stadler, 1995). Subsequent SRTT experiments have since explicitly investigated timing by testing whether temporal sequences (i.e., patterns of long and short inter-stimulus intervals) can be learned independently from ordinal (visual) sequences. Whereas some research varying the RSI suggests that temporal patterns are learned only when the ordinal sequence is also structured (Buchner & Steffens, 2001; O’Reilly et al., 2008; Shin & Ivry, 2002), these claims are complicated by the temporally uncontrolled nature of RSI. Indeed, in an ordinally unstructured, auditory version of the SRTT Brandon et al. (2012) used ISI (i.e., wherein stimuli are timed irrespective of the participant’s response) to demonstrate the implicit learning of complex temporal patterns. Moreover, Kornysheva et al. (2013) found that novel visual-ordinal sequences are learned faster if their ISI temporal sequence is familiar, in comparison to novel ordinal sequences with novel temporal structure, suggesting that temporal predictability may be advantageous even when the ordinal structure changes.

### Metre

The role of metre in sequential learning has primarily been explored by combining differing durations of ISI in auditory syllable identification tasks (Brandon et al., 2012; Schultz et al., 2013; Tillmann et al., 2011). Brandon et al. (2012), for example, used patterns of varying ISI to investigate whether participants could implicitly learn a complex temporal structure. The timing of stimuli produced the effect of rhythms with different underlying metres: one with a grouping of two, and the other with a grouping of three. Although implicit learning of the temporal sequence was demonstrated for both conditions, the authors discovered some differences in performance on the basis of the underlying metre (Brandon et al., 2012). Other work has compared implicit ISI rhythm learning with and without overt metric structures (Schultz et al., 2013), finding that metrical temporal patterns were not learned more readily than non-metrical temporal patterns. Finally, Terry et al. (2016) examined temporal sequence learning in the context of rhythms where the metric structure is less readily accessible to participants. They found that adding a regularly occurring (600 ms ISI) woodblock sound to compliment the presentation of syllable sounds decreased RT for only one of the two rhythms they investigated, which the authors speculated had a comparatively stronger sense of metre (Terry et al., 2016). Taken together, the results indicate that metre can affect performance in the sequential learning of temporal structure.

Whereas these temporal manipulations have thus far mostly been limited to absolute changes in the inter-stimulus or response-stimulus interval, the prospective role of metre and, in particular, accent when ISI are constant in the traditionally visual-ordinal SRTT remains under-specified. For example, Selchenkova et al. (2014a) found enhanced learning in an auditory artificial grammar paradigm for patterns of varying ISI that expressed an underlying metre, in comparison to a condition where ISI were isochronous. However, it is unclear whether using auditory *accent* in a visual task timed with isochronous ISI may also induce a sense of metre. Accents manifest in a variety of ways, some of which are mutually incompatible; in music, for instance, a musical tone that is longer or shorter, or lower or higher, than its neighbouring sequential elements may be perceived as accented. Speakers can increase the duration, intensity, or pitch of syllables to produce verbal accents (stress), which are achieved in sign language by analogously longer, more vigorous, or higher positioned movements (Wilbur & Schick, 1987). Runners optimise their stride by pacing steps to the “accent” of the initiation of the respiratory cycle (MacPherson et al., 2009). Put briefly, accent is a deviation from some recurring norm. Our sensitivity to accent may help us cope with our dynamic environment, by concentrating our attention and energy where it is most needed. Auditory targets co-occurring with accents are detected more quickly than those that occur at unaccented moments (Bolger et al., 2014; Cason & Schön, 2012), and performance in visual tasks may also be boosted when targets are paired to auditory accent (Escoffer et al., 2010; Trost et al., 2014). Vocal stress and emphatic gesture are shown to selectively enhance the learning and memory of concomitant semantic information (Biau & Soto-Faraco, 2013; Bozkurt et al., 2016; Igualada et al., 2017; Munhall et al., 2004). Even unattended background rhythms appear to induce a sense of metric expectancy, the violation of which can be detected in pupillometry (Damsma & van Rijn, 2017) and EEG (Geiser et al., 2010). Metric modulation of neural oscillatory phase and power has also been demonstrated (Fujioka et al., 2009; Iversen et al., 2009; Snyder & Large, 2005; Will & Berg, 2007), even when participants are simply asked to imagine subjective accents (Fujioka et al., 2014, 2015; Nozaradan et al., 2011; Vlek et al., 2011). Real or imagined, the cognitive implications of accent may extend to the dynamic allocation of attention; intuitively, it is plausible that temporally dynamic patterns should be more successfully learned via temporally dynamic attentional mechanisms, as is proposed to be true for language acquisition (de Diego-Balaguer et al., 2016).

### The current study

Taken together, the question arises whether metre and its potential to temporally modulate attention (Jones, 1987; Jones & Boltz, 1989) could play a role in sequence learning, even in a task with no explicitly metrical component, such as the standard SRTT. Hypothetically, such an effect would probably exhibit high inter-individual variability (Grahn & Schuit, 2012), but some groups may be more likely than others to respond to, or develop an internal sense of, metre. Musicians, for example, may have an increased sensitivity to metre due to their working knowledge of rhythm and overall superior timing skills (Kotz et al., 2018; Miendlarzewska & Trost, 2014). Even in the context of language, musicians’ behavioural and brain responses reveal a heightened sensitivity to metric structure, as well as its violation, in comparison with non-musicians (Marie et al., 2011). Musicians may have an advantage in the SRTT, due to enhanced sensory–motor mapping (Anaya et al., 2017) and sensitivity to statistical regularities (Romano Bergstrom et al., 2012). They also have superior coordination and fine motor skills attributable to years of practice (Schlaug, 2001; Stewart, 2008); hence, including musicians may be of advantage in detecting the potentially mediating effects of metre in the SRTT, noting that trained musicians tend to come from higher socioeconomic status groups and therefore possibly benefit from a range of enriching activities from early development onward (Amso et al., 2019). To alleviate this latter concern, we purposely avoided language, both in our recruitment materials and when collecting demographic information, that prioritised a Western classical model of musical expertise (e.g., measuring for how long one has studied harmonic theory or taken formal lessons), opting instead for a self-rating of active musical engagement.

This study makes several primary predictions: first, that metrical structure should modulate visual sequential learning due to metrically induced attentional fluctuations, such that visual elements coinciding with accented auditory elements will exhibit relatively shorter RT during learning (Large & Palmer, 2002). Second, the strength of this effect should vary among participants in respect to their individual abilities to detect and make use of metric information. A range of musical backgrounds were sought during participant recruitment, but as rhythmic aptitude is also known to vary within self-identified musician groups (Bailey & Penhune, 2010; Matthews et al., 2016), we separately tested all experimental participants in an independent auditory rhythm discrimination task and took their accuracy scores as a proxy of rhythmic sensitivity for covariate analysis in the SRTT. Finally, the third prediction is that, if sequential rhythm is encoded alongside sequential content (Bortoletto et al., 2011; Kornysheva et al., 2013), systematically altering the relationship between the learned auditory metre and learned visual sequence will exert an adverse effect on RT. It is possible that the putative mechanisms underlying these predictions may interact: for example, an individual’s susceptibility to metric influence could influence how well that person is able to make use of metric information when encoding sequential content (Jones, 2009; Selchenkova et al., 2014a, 2014b). In this case, we might expect to see that individuals who were better able to implicitly detect and integrate the auditory metre during learning (i.e., by producing faster RT to auditory accented-visual cues) will also be more adversely impacted when the correspondence between auditory metre and visual sequence is changed. In the case that cross-modal associations are not related to metric influences on attention at all, and are rather caused by the combining of ordinal information from the auditory pattern, with ordinal information from the visual pattern (Bouwer et al., 2016; O’Reilly et al., 2008), we should not necessarily expect to see a strong association with individual sensitivity to rhythm, nor a correspondence between metric patterns during learning and disruption under testing.

**Fig. 3:**
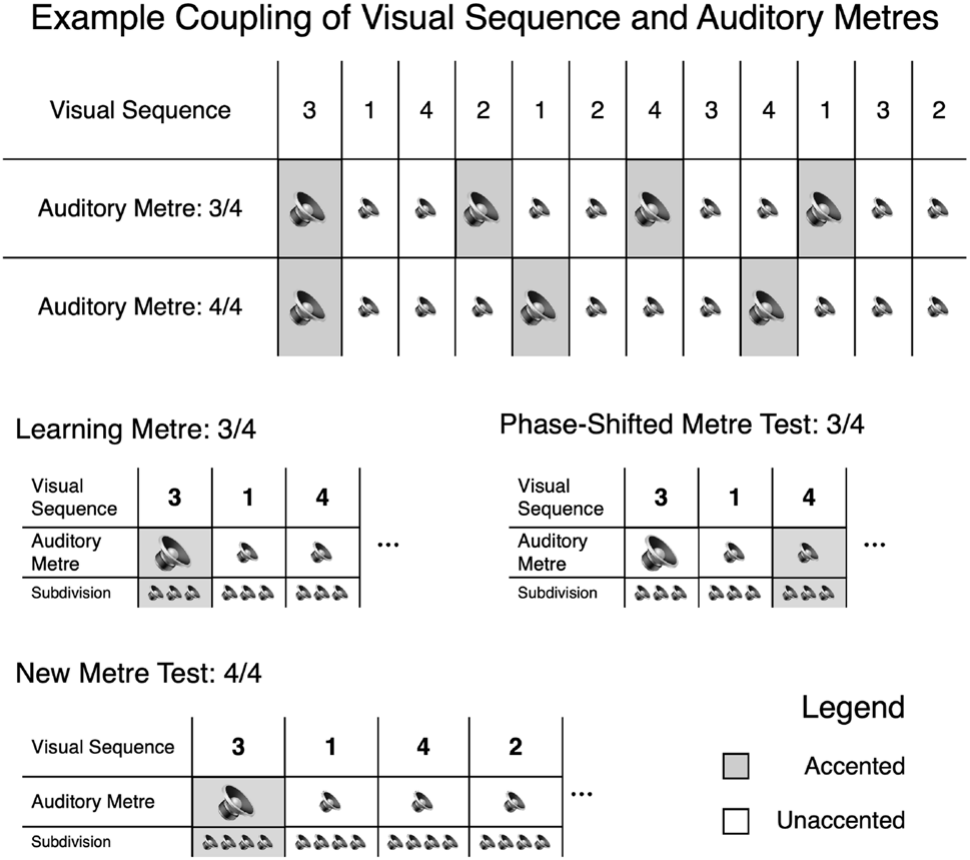
This figure depicts a graphical representation of possible pairings between a given visual sequence and the two auditory metre conditions, 3/4 and 4/4. In (**a**), an entire example sequence is shown in the upper row alongside the 3/4 m (middle row) and 4/4 m (bottom row). The same 12-element sequence can be paired with either metre. In the small lower panels, the main components of the serial reaction time task are presented. In (**b**), the learned metre is 3/4, and the same visual cues always coincide with the same position in the auditory metre. **c** shows that during the Phase-Shifted Metre Test block, the visual sequence and auditory metre each remain unchanged, but no longer correspond to one another as they did during learning. In (**d**) depicting the New Metre Test block, the visual sequence is again held constant, but the auditory metre condition changes, in this case, to a 4/4 pattern. Darker shaded boxes indicate accented auditory stimuli, and the rows labelled “Subdivision” depict the faster paced background rhythm to reinforce the sense of metre

We prepared a modified SRTT to impose a sense of metre via auditory rhythms consisting of a pattern of accented and unaccented drum sounds. Whereas timing in the form of non-isochronous inter-stimulus intervals has received attention in SRTT research [e.g., Brandon et al. (2012), Kornysheva et al. (2013), O’Reilly et al. (2008), Schultz et al. (2013), Shin & Ivry (2002)], the current project investigates the potential role of auditory metre using fixed inter-stimulus intervals in an otherwise canonical visuomotor paradigm. According to their randomly allocated condition, participants could hear a metre characterised by groupings of either three or four beats. Critically, although the auditory stimuli are always timed to coincide with the visual cues, they are ostensibly irrelevant to the task and no special direction is given concerning their presence to experimental participants, who are instructed only to respond as quickly as possible to the visual cues. We also introduced two metre-specific test blocks, wherein the familiar auditory metre has been altered: in the first of these test blocks, the pairing between the auditory metre and visual sequence undergoes a phase shift by one step, such that the accented and unaccented drum sounds no longer corresponded to the same visual cues as during the learning blocks; in the second, a new metre consisting of a novel pattern of accents was introduced in place of the familiar one (a schematic diagram of these task components is shown in Fig. 3). In both cases, the learned visual response locations are preserved, allowing us to ascertain whether the auditory metre is integrated during visual sequence learning, despite no overt indication to the participants that the cross-modal aspects of the task are consistently related to each other. The two metre test blocks differ both in terms of the nature of the interventions, as well as in their subtlety. Specifically, we hypothesised that the phase-shifted metre test was unlikely to be explicitly obvious to participants. Previous work in cross-modal implicit learning has employed auditory tone (i.e., pitched) stimuli (e.g., Hoffmann et al. (2001)), the sequencing of which form a melody that may be more accessible to participants, especially those with less musical experience, in comparison to the non-pitched drum sounds used in the current experiment. Hence, we should expect to see more of a negative impact of phase-shifting the metre on participants with higher sensitivity to rhythm, according to the rhythm discrimination task. We devised the New Metre test block as a more drastic intervention that would be likely to prompt conscious awareness in most participants. This should also perturb participants who may not have encoded the familiar auditory metre during learning, but who nonetheless are affected by its disturbance in the moment. In terms of the predicted magnitude of the effects, we expect to see slowing of RT in participants with greater sensitivity to rhythm in when the metre is phase-shifted, and more dramatically adverse effects on RT following the introduction of an entirely new metre.

## Method

### Participants

A total of 50 participants were recruited, with data from four individuals lost due to equipment error, resulting in *n* $= 46$ (22 male). Of those retained, 20 (43.48%) had been randomly allocated to the 3/4 m condition and 26 (56.52%) to the 4/4 m condition. The mean age was 21.6 years (SD 3.95), range 18-34. Participants varied in formal educational attainment, and reported a variety of first languages and cultural backgrounds, as well as musical experience ranging from little to no interest in music, to professional training. All participants were screened for normal to corrected vision and normal hearing, and reported no history of neurological disorders. The study was performed in line with the principles of the Declaration of Helsinki and received approval from departmental ethics committees. All participants gave informed, written consent prior to beginning the experiment, and were compensated for their time. Testing took place at University of Cambridge and University College London in the United Kingdom. At both testing locations, participants were seated in a quiet space in the presence of the experimenter, who discretely monitored task performance during a single experimental session of approximately 1 h in duration. The experiment was structured in the following order: (1) SRTT; (2) Rhythm Discrimination Task; (3) Musical Experience Questionnaire.

### Materials and procedure

#### Serial reaction time task

As per the canonical procedure, the visuomotor aspect of this task entailed participants responding as quickly as possible to each cue location in a graphic display by pressing its corresponding key (Z, X, N, or M) with their two index and middle fingers on a regular computer keyboard. The cue locations were deterministic, as probabilistic sequences substantially complicate the interpretation of temporal structure in SRTT responses (Schultz et al., 2013). Visual stimuli consisted of a grey background upon which white X-shapes appeared in one of four possible locations spanning horizontally across the screen at a fixed presentation rate of 1 Hz. The locations and serial order of response locations were determined by a 12-unit, equal-frequency, equal-transitional probability ordinal sequence. To counteract sequence- and key-specific effects, participants were randomly allocated to one of six different basic sequential structures (e.g., 3-1-4-2-1-2-4-3-4-1-3-2) and one of two spatial cue and key response mappings (e.g., 3-1-4-2 could be realised as either N-Z-M-X or Z-N-X-M), resulting in a total of 12 different sequences. In the current experiment, rhythm and metric structure were implied via task-irrelevant auditory stimuli. Each visual cue appeared simultaneously with the sounding of a drum tone to generate the sense of a musical beat, and the same visual cue always appeared with the same drum tone during learning. Performance was measured via reaction time (RT) in milliseconds. Participants were randomly allocated to one of two metric conditions: 3/4, which consists of one accented beat followed by two unaccented beats; and 4/4, which consists of one accented beat followed by three unaccented beats. Hence, the 12-unit visual sequence transpired over four cycles of 3/4, and three cycles of 4/4. Each of these two metres is represented in popular Western music; however, 4/4 is generally more common. Contrasting levels in accent were conveyed using naturalistic, non-pitched drum sounds, with a snare drum for accented and hi-hat for unaccented beats. Sixty beats per minute (1000 ms ISI) is a relatively slow tempo, and popular music is unlikely to have silence between tones or beats at this pace. We therefore included an isochronous, quieter background subdivision (a faster paced rhythm) to improve ecological validity in this regard, which was also used to reinforce the contrast between the 2 m. This was achieved by incorporating short, percussive sounds to divide the temporal space between each beat equally into three 333 ms segments for 3/4 (creating what is known as a 9/8 metre in musicological terms), and four 250 ms segments for 4/4.

The auditory rhythm stimuli were generated using recorded samples freely obtained online from MusicRadar (2009) and were edited in version 2.1.3 of Audacity software (Audacity Team, 2014). The drum sound used to imply accented beats is “Acoustic snare”; unaccented beats, “closed hihat”; and the background subdivision sound, “tick”. The sound intensities were adjusted for musical naturalness, but the metrical strength is implied by type of drum sound alone (i.e., not using intensity accents). Auditory stimuli were presented via Sennheiser HD 212 Pro studio headphones (Sennheiser Electronic Corporation, CT) set to a comfortable volume, adjusted individually for each participant. Task responses were recorded from the laptop’s keyboard directly.

Prior to beginning the SRTT proper, participants completed a practice session, which consisted of a visual sequence that was common to all conditions and paired to only unaccented drum sounds without any background subdivision sound. Participants received automatic text feedback on their accuracy and speed, with a prompt encouraging them to respond as quickly as possible for the rest of the task. Each block of the SRTT began at a different cue position within the auditory metre and visual sequence, and consisted of ten cycles equating to 120 cues per block, followed by a self-paced resting period before beginning the next round. The block structure was formally organised as follows:

1.–8. Eight Learning blocks, wherein participants saw a recurring visual sequence and heard a recurring auditory metre, which were always paired together in the same configuration.
9. The Phase-Shifted Metre block, wherein the visual sequence and the auditory metre were re-aligned, so that the learned spatial cues differently corresponded to the learned auditory accent pattern, e.g., a visual sequence location that was previously associated with an accented drum tone would instead co-occur with an unaccented drum tone during this test block.
10. A single Learning block, comprised of the learned visual sequence and learned auditory metre.
11. The New Metre block, in which case, participants who learned with the 4/4 m now instead heard the 3/4 m paired to their learned visual sequence, and vice versa.

The test blocks (blocks 9 and 11) were compared with RT from the immediately preceding late learning blocks (blocks 8 and 10, respectively). We confirmed with piloting (performed informally with 5 student volunteers unfamiliar with the study) that the transition from learning into phase-shifted metre was subtle, and unlikely to be consciously noticed by participants. In comparison, the New Metre block was obvious, even to musically untrained individuals. To preserve the low profile of phase-shifting the auditory metre, we therefore held the block order constant.

After completing the main SRTT, to ensure that the basic premise of visual sequential learning had been achieved, participants were presented with an unfamiliar visual sequence paired with the familiar auditory metre (*n* = 120). Responses from this check were compared to RT from pooled from Blocks 8 and 10 in the main task. A subset of participants who performed the main task (*n* = 26, 10 of whom were in the 3/4 m condition) were further tested for explicit knowledge of the learned SRTT sequence to confirm that our modified version of the task did not inadvertently encourage explicit learning strategies using a sequence completion task. Details of these checks are given in Online Appendix C.1.5.

*Rhythm discrimination task* To estimate individual participants’ perceptual sensitivity to auditory rhythm, a two forced-choice discrimination task was prepared. On a trial to trial basis, the objective was to determine whether a second rhythm differed from the first. The auditory stimuli consisted of 3.2-s rhythmic patterns introduced in Tierney & Kraus (2015), originally adapted from Povel & Essens (1985), which were formed by the arrangement of 9 percussive conga drum sounds separated by the following inter-onset intervals: 5 $\times$ 200-ms; 2 $\times$ 400-ms; 1 $\times$ 600-ms; and 1 $\times$ 800-ms (Tierney & Kraus, 2015), the re-ordering of which generate distinct rhythmic patterns. Each trial began with the presentation of a visual cue on the computer screen for 500 ms, followed by a a continuous presentation of two repetitions of an auditory rhythm. After a pause of 2000 ms, the visual cue reappeared, and a second rhythm was initiated, which did not repeat. After the second rhythm stopped playing, the participant was immediately prompted to enter their “same”/“different” response, and the next trial began after a random delay ranging from 2000 to 5000 ms. Before beginning the task proper, participants first completed a short practice session, which provided accuracy feedback following each practice trial (*n* = 3, with 2 “different” correct answers). No test trial feedback was provided. For “different” trials, the level of difficulty was varied by pairing rhythms that were more or less similar in terms of their pattern of inter-onset intervals, as determined by the first author, who is a professionally trained jazz musician and educator. For example, a “different” trial would be considered to be relatively difficult if both rhythms started with the same pattern of inter-onset intervals, but deviated by a slightly differing order of intervals within the second parts of the two rhythms. The task started with mostly easier “different” trials, before finishing with mostly harder “different” trials, with the intended goal of easing in participants who might feel less confident with such a task. Trials were equally distributed between “same”/“different” categories, and “different” trials were also equally distributed across “easier”/“more difficult”. The task consisted of forty trials with two self-paced rest periods. All participants heard the same trial order. The resultant dependent variable Rhythm Score was percent correct “same”/“different” responses, from a maximum score of 40.

*Musical Experience* Participants were asked to report, on a five-point Likert scale with 1 being “no experience” and 5 being “expert experience”, whether they currently, or had ever, played a musical instrument (including vocals). In a subset of the participants who also performed the SRTT (*n* = 16), we also collected the Goldsmiths Musical Sophistication Index (Gold-MSI), a psychometric tool for the measurement of musical attitudes, behaviours, and skills (Müllensiefen et al., 2014), and report in Online Appendix B correlations between rhythm sensitivity, as measured, and General Sophistication, as well as the Perceptual Abilities and Musical Training sub-scales.

*Analysis* The data were preprocessed in MATLAB (Mathworks, Natick, MA). Only correct SRTT trials > 50 ms were retained for further analysis. Raw or untransformed RT formed the dependent variable, but RT is generally plotted as mean-centred reaction times, which are calculated by removing each participant’s mean RT from their data points. Similarly, performance in the Rhythm Discrimination Task, hereafter termed Rhythm Score, was modelled as a continuous variable, but is sometimes also summarised as a binary low/high Rhythm Score group membership for plotting and descriptive analysis. These groups were calculated by taking the median Rhythm Score split. For the SRTT analysis, we identified the following main contrasts of interest:

1. Accented versus Unaccented responses during learning (Blocks 1–8).
2. Phase-Shifted Metre (Block 9) versus late learning (Block 8).
3. New Metre (Block 11) versus late learning (Block 10).

In the case of Contrast 1, predictors of interest included: Metre and Accent as factors; and Rhythm Score and Block (Blocks 1 - 8) as continuous variables. For Contrasts 2 and 3, predictors of interest included: Metre and Block; and Rhythm Score as a continuous variable. Random intercepts and slopes were estimated within Participant. Statistical tests were carried out using linear mixed models [LMM; lmer from the lme4 package (Bates et al., 2014) in R], after ensuring model residuals were normal via diagnostic procedures (DHARMA package Hartig (2019)). Although it has been recommended in the psychology literature to use generalised linear mixed effects models for reaction time data in general (Lo & Andrews, 2015), this advice should in fact only apply when the distribution of model residuals, and not of the raw data, is non-normal (Kéry & Hatfield, 2003). Model term selection was guided by Akaike information criterion [AIC;Sakamoto et al. (1986)]. *t*-Statistics with Satterthwaite degrees of freedom calculated the significance of fixed effects (car package (Fox et al., 2012)). For each analysis, full details concerning the model and its specification, including random intercepts and slopes, are reported in the Supplementary Materials. Significant interactions were further investigated using Tukey’s honestly significant difference (HSD) tests of estimated marginal means [emmeans, Lenth et al. (2018) in R]. The semi-partial $R^2$ (R$^2_{sp}$) is reported as an indication of relative magnitude of effect sizes (Jaeger et al., 2017). Confidence intervals for the regression coefficients and *R*$^2_{sp}$ were both calculated in R using Confint and R2beta (Jaeger et al., 2017), respectively. Other pairwise contrasts were conducted using *t*-tests (ttest2 in MATLAB) or Mann-Whitney U-tests (ranksum in MATLAB), and linear trends were inspected with Pearson’s linear correlation coefficient or Spearman’s rank coefficient (both corr in MATLAB). The threshold for statistical significance was 0.05 and multiple comparison corrections were applied to post hoc contrasts using the Bonferroni method. The false discovery rate (FDR; Benjamini-Hochberg procedure) was used to correct correlations and the permutation analyses in the exploratory section. In all cases, adjusted *p*-values are reported. Descriptive statistics (e.g., Mean, SD) reported at the group level are first aggregated within Participant.

## Results

### Summary statistics

We began by identifying possible anticipatory responses, wherein the correct key was pressed either $< 50$ ms after cue presentation, or just before the beginning of its response period (i.e., while the previous cue was still displayed). A total of 5 participants (*N*_3/4 m_ $= 3$, *N*_4/4 m_ $= 2$) produced more than 10% correct anticipatory responses across multiple blocks and were excluded from further analysis, resulting in a final *n* of 41 (3/4 m condition $= 17, 41.5\%$, 4/4 m condition $= 24, 58.5\%$). The remaining correct anticipatory responses (mean $= 2.24\%$, SD $= 1.84\%$) produced by other participants were removed. Overall, there were 49,785 (93%) correct responses retained. Mean percent correct did not vary by Metre condition, *U*(*N*_3/4 m_ $= 17$, N_4/4 m_ $= 24$) = 347.5, *z* = – 0.24, *p* $= 0.81$) and did not correlate with Rhythm Score (*r*_s_ $= 0.01$, *p* $= 0.94$). Calculated within participants (41), the mean of mean RT was 430.98 ms, 95% CI [401.23,460.74], SD = 94.27, Range = [247.01,833.66]. A visualisation of the complete task is shown in Fig. 4a. Descriptive statistics concerning self-reported musical background and performance in the Rhythm Discrimination Task are given in Table 1, the measures of which were correlated, *r*$_\mathrm{s} = 0.65$, CI [0.43, 0.79], *p* $< 0.001$. Median levels of instrument playing did not statistically differ between the 3/4 m (median $=$ 2.5, IQR $=$ 2.5) and 4/4 m (median $=$ 2, IQR $=$ 2) conditions, *U*(*N*_3/4 m_ $= 17$, N_4/4 m_ $= 24$) = 310, *z* = -0.05, *p* $= 0.96$. Rhythm Score, defined as percent correct in the Rhythm Discrimination Task, ranged from 42.5 to 100, with a median score of 85. As regards the distribution of Rhythm Score across Metre conditions, the 3/4 condition (median $=$ 87.5, IQR $=$ 15.38) did not significantly differ in comparison with the 4/4 condition (median $=$ 83.75, IQR $=$ 22.5), *U*(N_3/4 m_ $= 17$, *N*_4/4 m_ $= 24$) = 390.5, *z* = 0.88, *p* = 0.38.

**Table 1:** Participant self-reported rating of musical experience on a 5-point Likert scale with 1 being “no experience” and 5 being “expert experience”

Self-reported musical experience				
Rating	Count	Percent	Rhythm Score	
			Mean	SD
1	4	10.5	66.88	18.19
2	15	39.48	77.5	12.85
3	7	18.42	79.29	18.47
4	8	21.05	87.5	6.55
5	4	10.5	92.5	7.91

### Planned contrasts

**Fig. 4:**
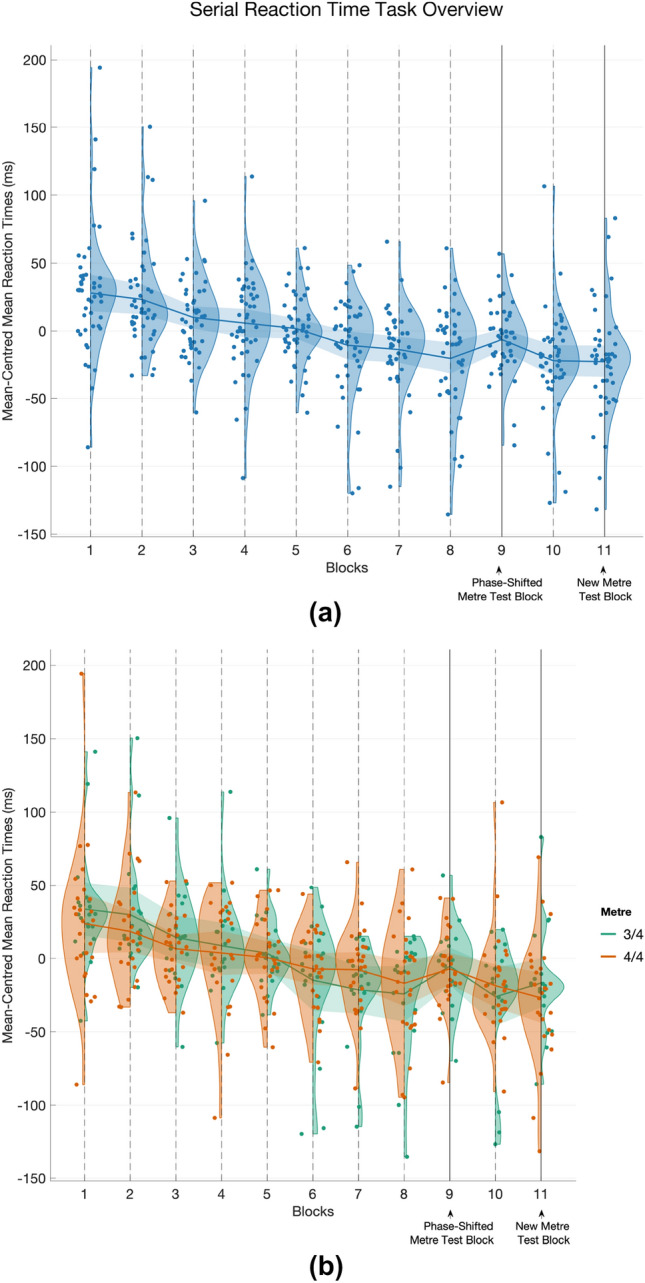
Overview of the the Serial Reaction Time Task showing mean-centred mean reaction times (calculated within participant). Blocks 1–8 and 10 are Learning blocks; Block 9 is the Phase-Shifted Metre Test; and Block 11 is the New Metre Test. Group means are shown by lines, with shaded region representing 95% confidence intervals of the mean. Upper panel (**a**): Full task. Lower panel (**b**): Grouped by Metre condition

#### Effect of accent during learning

**Fig. 5:**
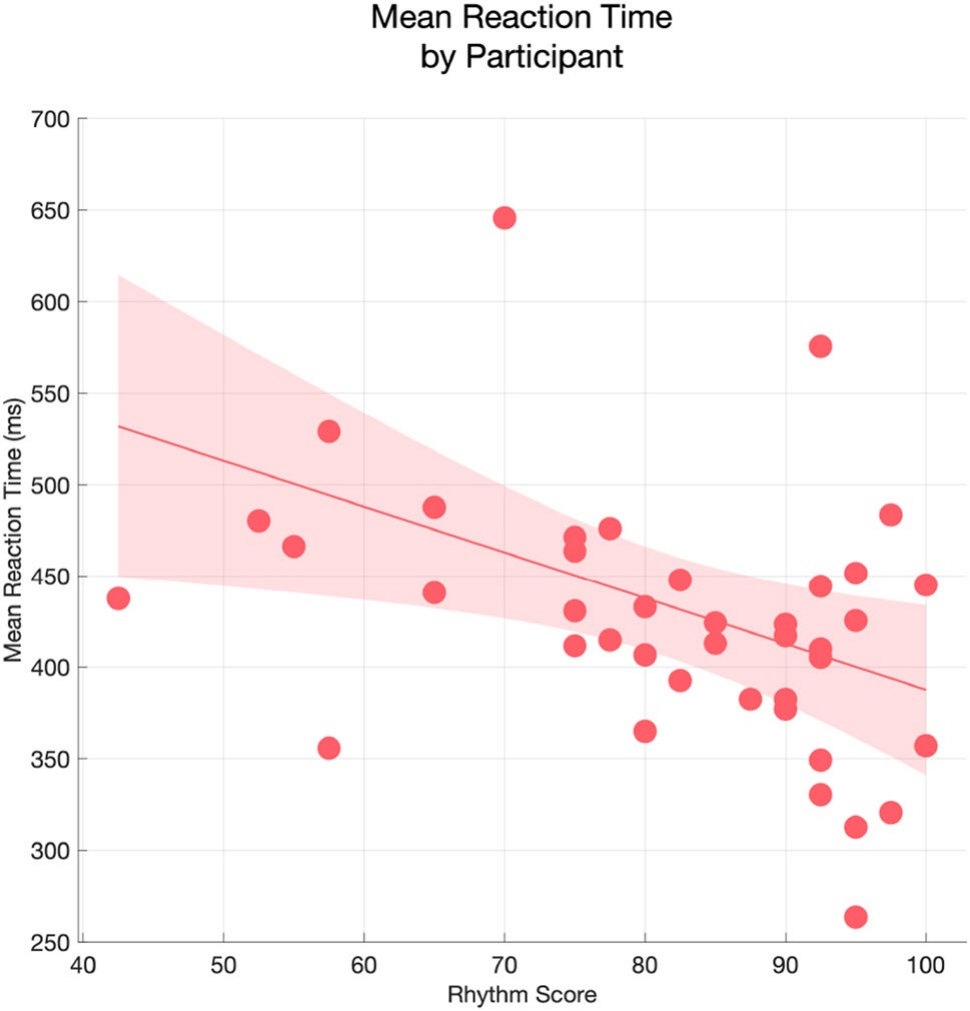
Mean reaction times (ms) as a function of Rhythm Score. Group mean is shown by a line, with shaded regions representing 95% confidence intervals of the mean

We hypothesised that, over the learning period (Blocks 1–8), visual cues associated with Accented auditory stimuli in the auditory Metre would elicit shorter RT. As a main effect, higher Rhythm Score was significantly predictive of faster RT (Estimate = – 38.28, 95% CI [– 65.95,– 10.6], *t*(1,37.99)= – 2.71, *p* $= 0.01$, *R*$^2_\mathrm{sp} = 0.084$), with a correlation between mean RT (calculated across Learning blocks) and Rhythm Score of $\rho _\mathrm{s}$ = – 0.44 (95% CI [– 0.55,– 0.08], *p* $= 0.004$; Fig. 5). Participants’ RT also grew shorter by Block (Estimate = – 8.28, 95% CI [– 12.42,– 4.13], *t*(1,42.42)= – 3.91, *p* $< 0.001$, *R*$^2_\mathrm{sp} = 0.003$), but there was also a two-way interaction between Accent and Metre (Estimate = 21.15, 95% CI [12.49,29.80], *t*(1,36018.83)= 4.79, *p* $< 0.001$, *R*$^2_\mathrm{sp}$ $\le 0.001$), and a three-way interaction between Block, Accent, and Metre (Estimate = – 2.65, 95% CI [– 4.36,– 0.95], *t*(1,36018.70)= – 3.05, *p* $= 0.002$, *R*$^2_\mathrm{sp}$ $< 0.001$). There was no main effect for Accent (*p* $= 0.17$), and the main effect of Metre fell short of statistical significance (Estimate = – 62.13, 95% CI [– 62.13,0.04], *t*(1,39.37)= -1.96, *p* $= 0.06$, *R*$^2_\mathrm{sp}$ $= 0.004$), although participants in the 3/4 m tended to be overall a little slower (Count = 17, Mean = 446.17, 95% CI [379.91,512.44], SD = 128.88) and more variable than in the 4/4 m (Count = 24, Mean = 429.71, 95% CI [403.17,456.25], SD = 62.85).

Post hoc contrasts revealed that responses significantly differed on the basis of Accent for participants in the 4/4 m condition (Estimate = – 6.52, SE = 1.34, *t*(1,36018)= – 4.88, *p* $< 0.001$), with a non-significant effect of Accent for participants in the 3/4 m condition (Estimate = 2.63, SE = 1.47, *t*(1,36018)= – 1.79, *p* $= 0.07$). Comparing mean RT across conditions, the 4/4 m participants tended to respond more quickly to accented-visual cues (Mean = 425.06, 95% CI [398.27,451.86], SD = 63.45) than Unaccented cues (Mean = 431.37, 95% CI [403.79,458.96], SD = 65.33). In contrast, for the 3/4 m participants, Mean Accented RT = 447.65 (95% CI [380.34,514.96], SD = 130.92) did not differ from Unaccented RT (Mean = 445.43, 95% CI [379.56,511.30], SD = 128.12).

**Fig. 6:**
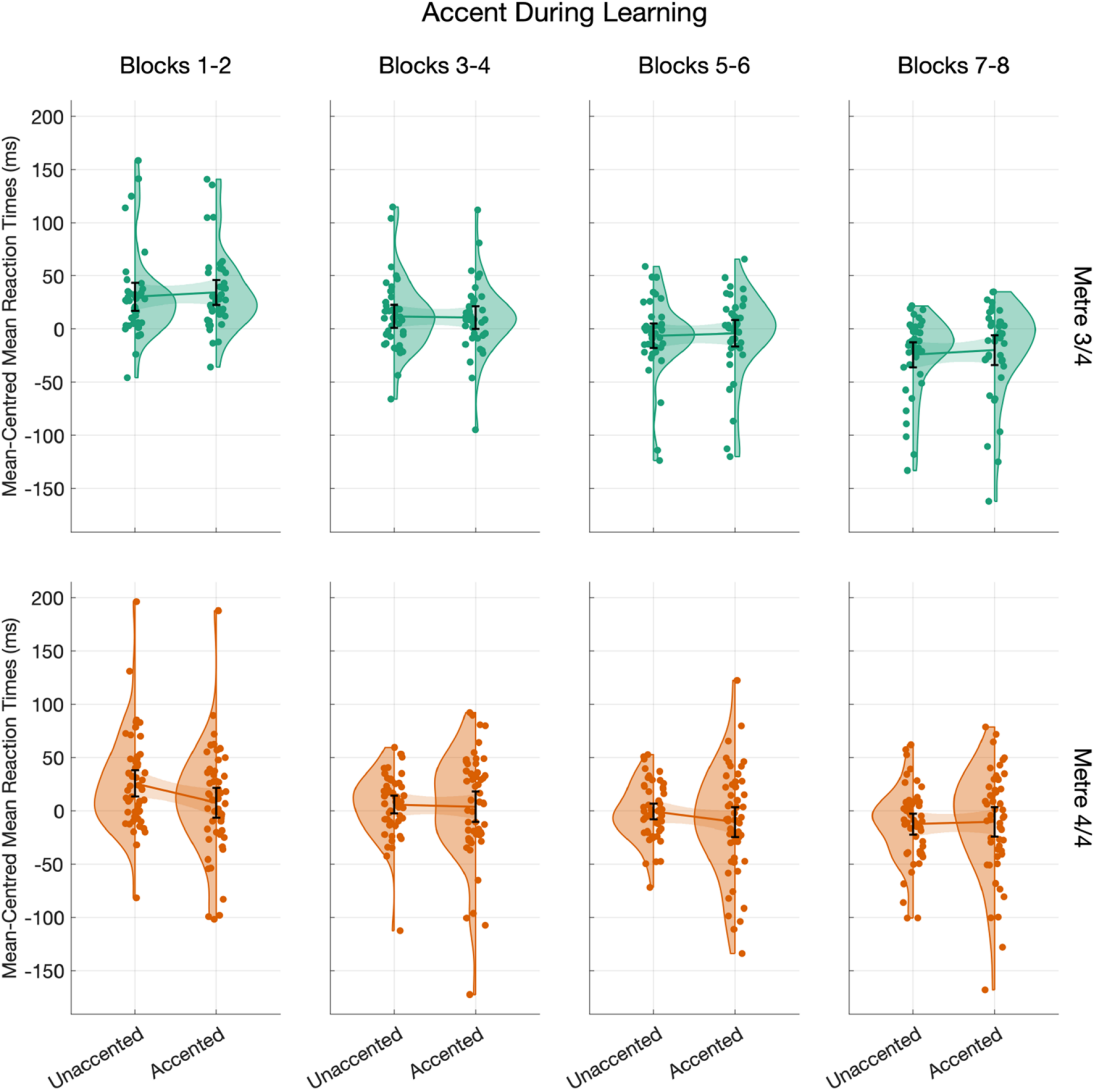
Mean-centred mean reaction times (calculated within participant) by Accent during Learning. The data are summarised by Metre across rows, and Block across columns (note that Blocks were modelled separately and are grouped by two here for simplicity). Group means are shown by lines, with the error bars and shaded regions representing 95% confidence intervals of the mean

This interaction between Metre and Accent was further mediated by Block, which was modelled as a continuous variable but is summarised here by groupings of two (e.g., Blocks 1/2) for visual and descriptive parsimony. For participants who heard the 3/4 m, their RT significantly decreased by Block for both Accented (Trend = – 8.26, SE = 2.12, *p* $= 0.001$) and Unaccented responses (Trend = – 7.86, SE = 2.08, *p* $= 0.002$). In the case of participants who heard the 4/4 m, however, only responses to Unaccented cues grew significantly shorter by Block (Trend = – 4.96, SE = 1.75, *p* $= 0.03$). The effect of Block was in comparison reduced and statistically non-significant for Accented cues in the 4/4 m (Trend = – 2.72, SE = 1.80, *p* $= 0.55$). Looking at changes in mean RT within the 4/4 m group, we find that Unaccented responses drop by 33.91 ms between Blocks 1/2 (Mean = 451.97, 95% CI [428.46,475.47], SD = 80.94) and Blocks 7/8 (Mean = 418.06, 95% CI [396.14,439.99], SD = 75.50). By comparison, Accented responses are only reduced by 13.39 ms from Blocks 1/2 (Mean = 435.26, 95% CI [412.13,458.39], SD = 79.66) to Blocks 7/8 (Mean = 421.87, 95% CI [399.69,444.05], SD = 76.39). In other words, Accented responses appear to have started from a faster baseline than Unaccented responses for the 4/4 m, and so the benefit of increased exposure by Block is reduced. These contrasts are summarised in Fig. 6. Full model details are given in Supplementary Materials in Appendix C, Table C2, and details on the post hoc analysis in Supplementary Materials in Appendix C, Table C3.

#### Phase-shifted metre test block

After the first eight blocks of learning had taken place, we systematically altered the relationship between the learned auditory Metre and learned visual sequence, by phase-shifting the pattern of Accented and Unaccented drum sounds (Block 9). Hence, although participants heard the same auditory Metre and saw the same visual cues as in earlier blocks, the sequences no longer corresponded in the same way. We predicted that this would lead to a slowing of RT in comparison to learning blocks, particularly for participants who scored higher in the Rhythm Discrimination Task.

There was a significant main effect of Block (Estimate = 6.47, 95% CI [1.02,11.93], *t*(1,9078.05)= 2.33, *p* $= 0.02$, *R*$^2_\mathrm{sp}$ $\le 0.001$), indicating that participants in general slowed between Block 8, a Learning block (Mean = 418.75, 95% CI [385.45,452.06], SD = 105.53) and Block 9, the Phase-Shifted Metre test block (Mean = 423.63, 95% CI [393.63,453.64], SD = 95.06). There was no main effect of Metre (*p* $= 0.31$), and although higher Rhythm Score was associated with shorter RT (Estimate = – 73.93, 95% CI [– 127.82,– 20.03], *t*(1,37.29)= – 2.69, *p* $= 0.01$, *R*$^2_\mathrm{sp}$ $= 0.045$), it was also involved in a significant three-way interaction with Metre and Block.

Post hoc analyses revealed that, holding Rhythm Score constant, the effect of the Phase-Shifted Metre test was statistically significant for the 3/4 m condition (Estimate = – 6.47, SE = 2.78, *t*(1,9078)= -2.33, *p* $= 0.02$) but not for the 4/4 m condition (Estimate = – 3.84, SE = 2.27, *t*(1,9078)= – 1.69, *p* $= 0.09$). Moreover, Rhythm Score predicted faster RT in the 3/4 m in Block 8 (Trend = – 5.20, SE = 1.93, *p* $= 0.04$) but failed to meet significance in Block 9 (Trend = – 4.58, SE = 1.93, *p* $= 0.09$). For the 4/4 m condition, the effect of Rhythm Score was non-significant across both Block 8 (Trend = – 1.97, SE = 1.22, *p* $= 0.45$) and Block 9 (Trend = – 1.54, SE = 1.22, *p* $= 0.85$).

**Fig. 7:**
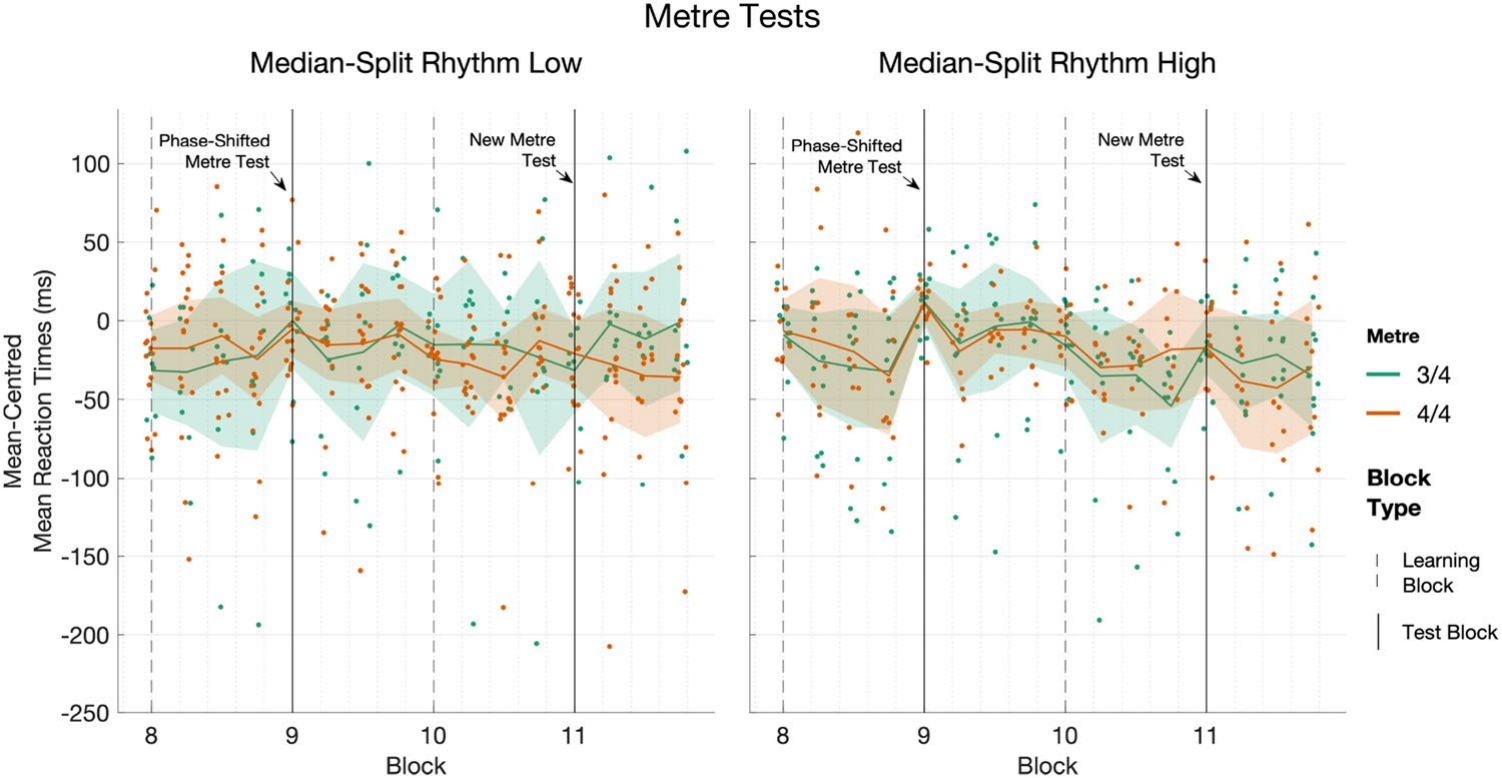
Overview of the the Metre Test Blocks showing mean-centred mean reaction times (calculated within participant). Blocks 8 and 10 are Learning blocks; Block 9 is the Phase-Shifted Metre Test; and Block 11 is the New Metre Test. The data are binned into quarter-blocks (*n* = 30)

It is possible that participants recovered to faster RT within the test block, so we looked at responses directly adjacent to the block boundaries (for details, see Supplementary Materials in Appendix A). In short, we compared the final two cycles of the sequence in Block 8, to the first two cycles of the sequence in Block 9, the test block. Summarising by median-split Rhythm Score group, there was a substantial overall increase in RT for participants in the High Rhythm group (mean of mean differences = +41.16, 95% CI [14.34,67.99], SD = 55.66) but less so for the Low Rhythm group (mean of mean differences = +2.34, 95% CI [– 17.16,21.83], SD = 43.97). Taken together, given that Rhythm Score is generally associated with faster RT throughout the task, and that the linear trend of Rhythm Score is overall reduced in Block 9 (Phase-shifted metre test) in the modelling, it appears that higher Rhythm Scoring participants were disproportionately affected by the change in association between the auditory metre and visual sequence. Full model details are given in Supplementary Materials in Appendix C, Table C4. An overview of the Metre Tests is depicted in Fig. 7.

#### New metre test block

In the following test block, the New Metre Test (Block 11), participants in the 3/4 m condition were exposed to a familiar visual sequence paired to the unfamiliar 4/4 auditory Metre, and vice versa. As with the previous Phase-Shifted Metre Test Block, we expected that disrupting the learned association between the familiar auditory Metre and visual sequence would result in relatively slower RT. We also predicted that we would see a more robust slowing in RT in comparison to the Phase-Shifted Metre Test block, because we expected that the effect of changing the Metre would be highly jarring, even for participants who had not integrated the auditory metre along with the visual sequence during learning.

There was no main effect of Block (*p* $= 0.63$), nor of Metre (*p* $= 0.30$), although higher Rhythm Score was again predictive of shorter RT overall (Estimate = – 90.59, 95% CI [– 144.64,– 36.54], *t*(1,37.32)= – 3.28, *p* $= 0.002$, *R*$^2_\mathrm{sp}$ $= 0.063$). There were also significant interactions, including a three-way interaction between Rhythm Score, Metre, and Block (Estimate = 71.09, 95% CI [7.24,134.95], *t*(1,37.31)= 2.18, *p* $= 0.04$, *R*$^2_{sp}$ $= 0.029$). Post hoc contrasts investigating this interaction indicated that, with Rhythm Score held constant, participants in the 3/4 m condition did not significantly change their RT after hearing the New Metre (Estimate = $-1.41$, SE = 2.96, *t* = – 0.48, *p* $= 0.63$). Within the 3/4 m, the reduction of the effect of Rhythm Score from Block 10 (Estimate = $-6.36$, SE = 1.94, *t* = – 3.29, *p* $= 0.008$) to Block 11 (Estimate = $-5.55$, SE = 1.94, *t* = – 2.87 *p* $= 0.03$) indicates that participants with higher Rhythm Scores may have been a little more likely to slow in association with hearing the New Metre.

In comparison, the results for the 4/4 m participants are more surprising: with Rhythm Score held constant, participants in the 4/4 m actually seemed to have significantly *increased* in speed after hearing their New Metre (Estimate = 10.92, SE = 2.45, *t* = 4.46, *p* $< 0.001$). Moreover, for the 4/4 m, we do not find any predictive effect of Rhythm Score in Block 10 (Estimate = $-1.37$, SE = 1.22, *t* = – 1.12, *p* $= 1.0$), nor in Block 11 (Estimate = $-1.63$, SE = 1.22, *t* = 37.3, *p* $= 0.75$). Breaking down the 4/4 m by Rhythm Score group, we find that the RT of Low Rhythm Score participants were similar between Block 10 (Mean = 425.20, 95% CI [394.07,456.34], SD = 53.93) and Block 11 (Mean = 423.04, 95% CI [393.18,452.89], SD = 51.71). However, the High Rhythm Score participants in the 4/4 m showed a different pattern of results, with their RT tending to improve between Block 10 (Mean = 388.88, 95% CI [304.64,473.11], SD = 117.76) and Block 11 (Mean = 365.64, 95% CI [323.19,408.08], SD = 59.33). Full model details are given in Supplementary Materials in Appendix C, Table C6. As we had done with the Phase-Shifted Metre Test, we compared RT during the final two cycles of the sequence in Block 10 with the first two cycles of Block 11, to ensure that a possible local effect of the New Metre Test was not obscured by pooling responses on the level of Block, but the pattern of results was the same (for details, see Supplementary Materials in Appendix A)

Because we had incorrectly predicted that the effect of the New Metre Test would be more consistently negative and of a greater magnitude than the Phase-Shifted Metre Test, we did not experimentally vary Block order, meaning that the learning block (Block 10) to which we compare the New Metre Test Block (Block 11) could potentially be influenced by the earlier Phase-Shifted Metre Test (Block 9). We therefore modelled responses between Block 8 and Block 10. We saw no main effects of Metre nor of Block (both *p* $> 0.29$), but there was an interaction between Rhythm Score, Metre, and Block (Estimate = 70.39, 95% CI [2.70,138.08], *t*(1,37.26)= 2.04, *p* $= 0.05$, *R*$^2_\mathrm{sp}$ $\le 0.001$). Upon closer inspection, however, we found this effect to be of the same general pattern as in earlier blocks, where the linear trend of higher Rhythm Score was predictive of shorter RT moreso for the 3/4 m in Blocks 8 and 10 (both *p* $< 0.03$) than for the 4/4 m in Blocks 8 and 10 (both *p* $> 0.12$). Importantly, neither the contrast between Block 8 and Block 10 (both *p* $> 0.32$), nor between the 3/4 m and 4/4 m (both *p* $> 0.29$) were statistically significant or otherwise indicative of a meaningful change that could be attributable to the intermediate Phase-Shifted Metre Test (Block 9).

It is possible that the Phase-Shifted Metre had some other downstream consequence that could have dampened the effect of the New Metre Test. However, given that participants in the 4/4 condition were slightly faster in Block 10 (Mean = 413.94, 95% CI [378.79,449.09], SD = 111.36) than in Block 8 (Mean = 418.75, 95% CI [385.45,452.06], SD = 105.53), and theoretically had more to lose, it seems less likely that this occurred. Alternatively, we could also speculate that participants were primed by the earlier test block, meaning that they were prepared for the New Metre Test. We cannot say for certain this did not occur, but during verbal debriefing, we found that no participants reported any notice or hint of the Phase-Shifted Metre test, despite most commenting on the New Metre Test. Hence, if any priming effect did occur, it was possibly unconscious. Taken together, although we cannot definitively establish that the Block order did not preclude the predicted effect of the New Metre Test, we can say that it was not because RT in the Learning Block 10 had yet to rebound from the Phase-Shifted Metre Test.

#### Implicit and explicit knowledge checks

After finishing the main SRTT, wherein the order of visual cues was held constant throughout both learning and the Metre test blocks, participants were exposed to an unfamiliar visual sequence, with auditory Metre held constant from the main task. Each participant’s RT from the new visual sequence test were compared with their RT from late learning (pooled Blocks 8 and 10) in the SRTT to verify that implicit learning of the visual sequence had indeed taken place.

**Fig. 8:**
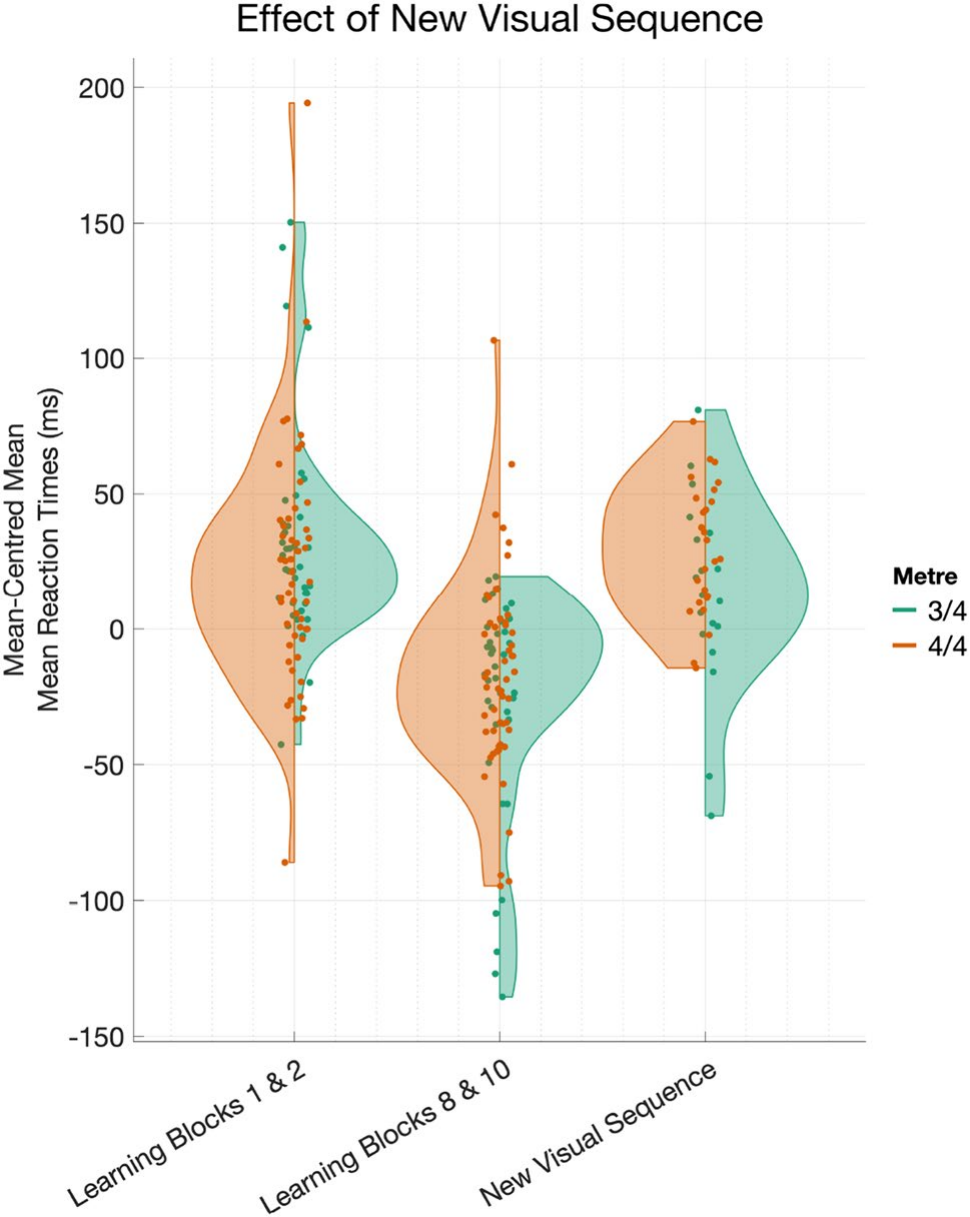
Mean-centred mean reaction times (calculated within participant) during Early Learning (Blocks 1 and 2), Late Learning (Blocks 8 and 10), and the New Visual Sequence Test, wherein the learned Visual Sequence was changed, but the learned Auditory Metre was the same as during Learning. Responses are summarised by Metre Condition

There was a significant main effect of New Visual Sequence (Estimate = 26.58, 95% CI [20.94,32.21], *t*(1,12539.33)= 9.24, *p* $< 0.001$, *R*$^2_\mathrm{sp}$ $= 0.003$), showing that participants’ responses to the changed visual sequence (Mean = 454.35, 95% CI [426.40,482.30], SD = 87.40) slowed by about 38 ms on average, in comparison to the final two learning blocks of the main SRTT (Mean = 416.54, 95% CI [383.15,449.92], SD = 105.78). This establishes that our modified, audiovisual SRTT functioned similarly to the canonical version of the task, in that participants appeared to have implicitly learned the visual sequence (Fig. 8). There was no main effect of Metre (*p* $= 0.31$), but holding Rhythm Score constant, we see that the magnitude of the slowing of RT is stronger for participants in the 4/4 m (Estimate = – 44.70, SE = 2.24, *t*(1,12537)= – 19.98, *p* $< 0.001$) in comparison with participants in the 3/4 m (Estimate = – 26.60, SE = 2.88, *t*(1,12539)= – 9.24, *p* $= 0.09$). Full details are given in Supplementary Materials in Appendix C, Tables C10 and C11.

To gauge explicit awareness of the SRTT learned sequence, we also administered an explicit recognition task wherein a subset of participants who performed the main task (*n* $= 26$) were also asked to guess the future location of a visual cue after viewing a segment of either two or three cues. Some segments were unfamiliar, and others in fact formed part of the participant’s learned sequence. As stated previously, participants who exceeded a mean of 10% anticipatory correct key presses were removed from the SRTT analysis. We ran the analysis of the explicit recognition task with and without (*n* $= 24$) these participants, and the overall pattern of results was the same. We report here statistics for the *n* $= 24$ participants whose data were also retained in the main SRTT, but details concerning both models are available in Supplementary Materials in Appendix C, Tables C12 and C13.

**Fig. 9:**
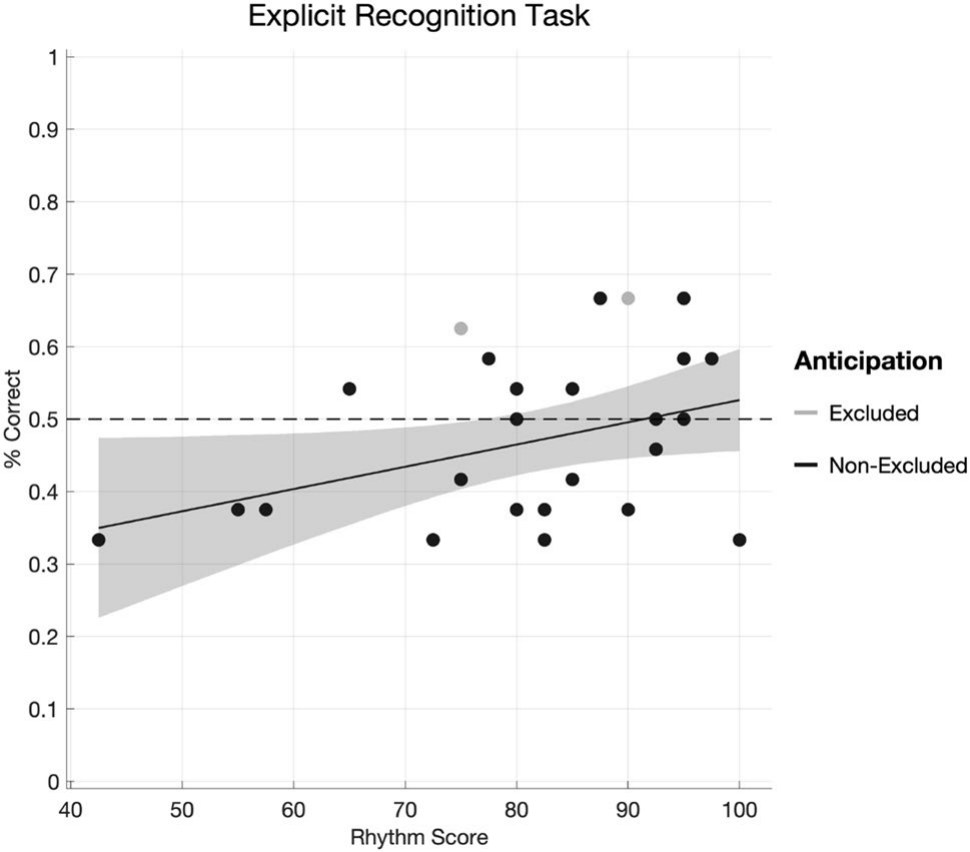
Mean percent correct (calculated within participant, task-wise) in the Explicit Recognition Task as a function of Rhythm Score. Lighter shaded dots indicate participants whose data were excluded from the serial reaction time task due to high ($> 10\%$) rates of correctly anticipated ($< 50$ ms) reaction times. Shaded regions represent 95% confidence intervals of the mean

The overall percent correct was 49% (95% CI [45, 53]). There was no improvement to models fit with Metre condition, self-reported knowledge of the visual cue, nor whether or not the number of cues shown matched the participant’s own SRTT Metre condition, as guided by AIC (range 802–806). There was a marginal main effect of Rhythm Score (Estimate = – 0.17, 95% CI $[-0.34,-0.00$, $\chi ^2 = 3.93$, *p* $= 0.05$, *R*$^2_\mathrm{sp} = 0.007$), but the Spearman’s rank coefficient between Rhythm Score and percent correct guesses (within participant) was weak and statistically insignificant, *r*$_\mathrm{s} = 0.22$, CI [0.09, 0.22], *p* $= 0.09$. Summarised by median-split Rhythm Score group, high rhythm scoring participants were correct 54% (95% CI [48, 59]) of the time on average, and low rhythm scoring participants did more poorly at 45% (95% CI [39, 51]). In general, although having a higher Rhythm Score may be associated with a negligible advantage for recognition, it appears that participants were overall unlikely to have acquired extensive explicit knowledge of their learned SRTT sequence. Mean correct guesses, including for the two excluded “anticipator” participants, are plotted by Rhythm Score in Fig. 9

#### Sensitivity to rhythm as an individual difference

Rhythm Score positively correlated with self-reported musical activity levels (1, “no experience” - 5, “expert experience”), *r*$_{s} = 0.65$, CI [0.43, 0.79], *p* $< 0.001$. We saw in our planned contrasts that higher Rhythm Score was associated with lower RT, but self-reported instrument playing and mean RT were also statistically related, *r*$_\mathrm{s} = -0.45$, CI $[-0.15, -0.66]$, *p* $= 0.004$, begging the question of whether Rhythm Score would predict RT in a non-musician subset (*n* $= 25$), which we defined as participants who self-reported musical activity $< 3$ on the 5-point Likert rating scale. Indeed, among non-musicians, Rhythm Score was still associated with shorter RT, *r*$_{s} = -0.43$, CI $[-0.74, 0.05]$, *p* $= 0.05$. In the musician subset (*n* $= 16$); however, there was no relationship, *r*$_\mathrm{s} = -0.04$, CI $[-0.59, 0.56]$, *p* $= 0.87$. Note that this may be an issue of this post hoc sample size, as Rhythm Score was nonetheless associated with lower mean RT across the complete sample, *r*$_\mathrm{s} = -0.44$, CI $[-0.1, -0.69]$, *p* $= 0.01$ (*p*-values FDR-corrected).

### Exploratory analysis concerning the effect of accent during learning

### Permutation tests

**Fig. 10:**
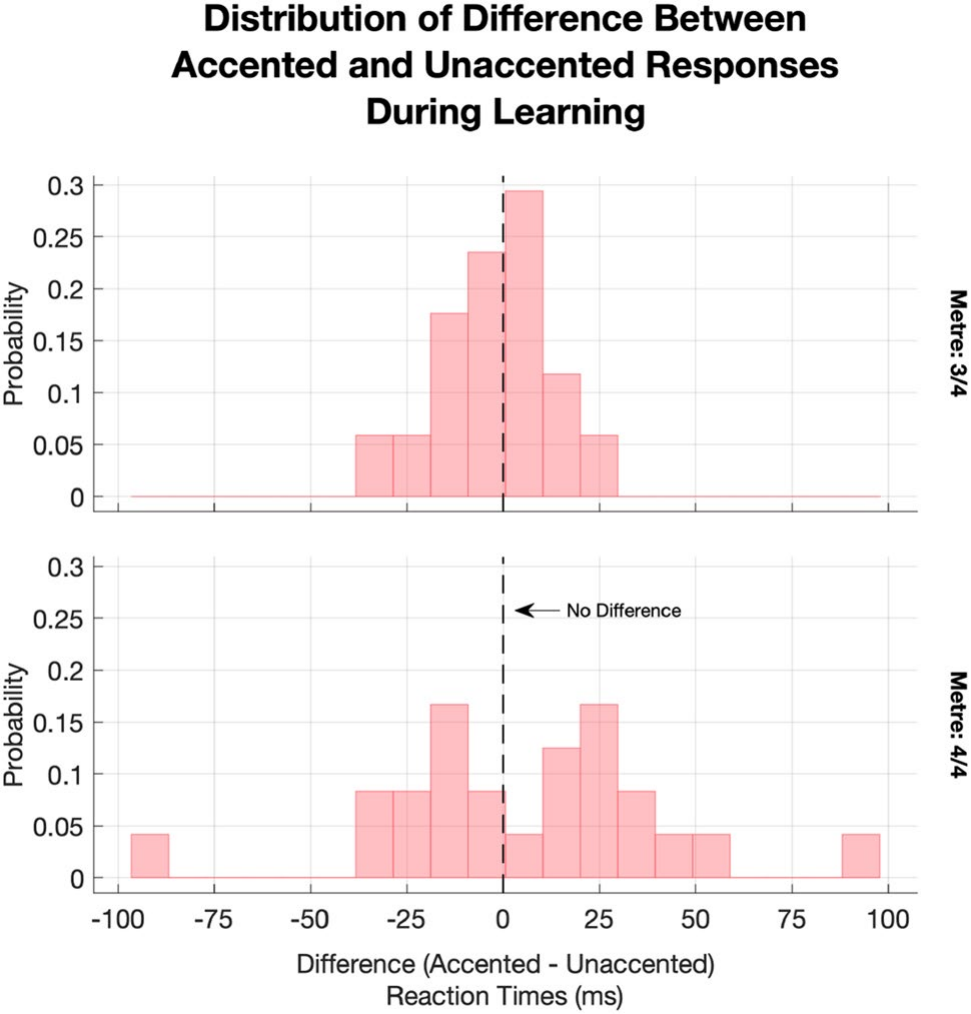
Histograms for the subtraction of Accented reaction times from Unaccented reaction times (ms), by Metre, during learning. Whereas the slight tendency for the 3/4 m towards slower Accented reaction times can be seen in the upper panel, the lower panel shows a possible bi-modal distribution of responses for the 4/4 condition

In our planned analysis of the SRTT, we established that participants in the 4/4 m condition responded more quickly to accented-visual cues during learning (blocks 1–8), and that participants in the 3/4 m condition showed a more reduced response in the opposite direction. Although these results were partly in line with our hypothesis, the effect was also subject to individual variability, especially during later Learning blocks. Visualising these individual differences, we became aware that, in the 4/4 condition, participants’ responses to Accent appeared to follow a bi-modal distribution (Figs. 6 and 10). Specifically, in addition to the expected peaks in Unaccented − Accented RT difference around 0 (participants who did not distinguish between Accented and Unaccented cues) and to the right of 0 (participants who responded more quickly to Accented cues), we also found a peak to the left of 0, indicating that some participants may have been systematically responding more *slowly* to Accented cues, resulting in potentially qualitative individual differences.

To verify this unanticipated pattern of results, we first conducted a simulated permutation test (*n* iterations $= 10,000$) by randomly shuffling each participant’s responses during learning, within response Key, within Block, and across the Accented and Unaccented cue conditions. We then calculated an Accent relative difference for each shuffled set, by taking the absolute difference between mean Accented RT $\bar{x}_\mathrm{Accented}$ and mean Unaccented RT $\bar{x}_\mathrm{Unaccented}$, divided by their arithmetic mean

1 $$\begin{aligned} \mathrm{Accent\,relative\,difference} = \frac{\left|\bar{x}_\mathrm{Accented} - \bar{x}_\mathrm{Unaccented}\right|}{\left( \frac{|\bar{x}_\mathrm{Accented} + \bar{x}_\mathrm{Unaccented}|}{2}\right) }. \end{aligned}$$

**Fig. 11:**
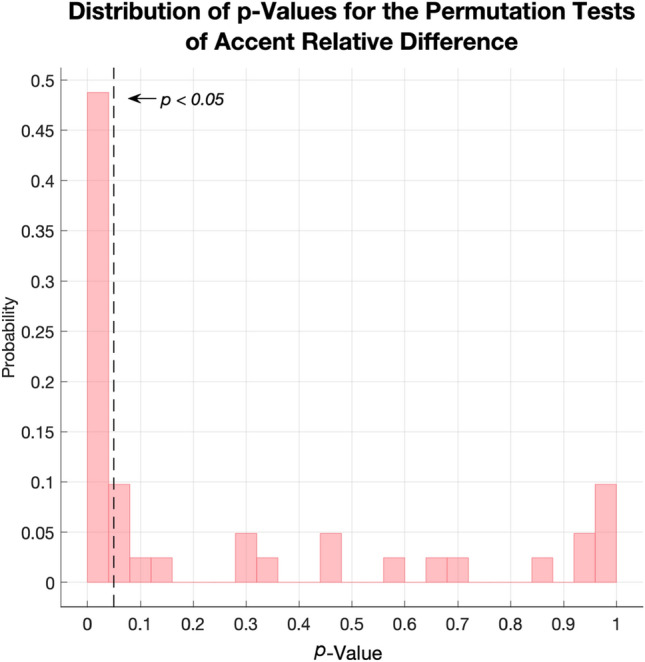
Histogram for the *p* values associated with individual permutation tests (*n*$= 10,000$), by participant, of mean Accent relative difference during learning. Tests shuffled responses within Key and Block. The dashed line represents an alpha level of 0.05 (false discovery rate corrected)

The Accent relative difference conveys the extent to which the participant’s Accented and Unaccented responses differ from another, irrespective of the direction or sign of that difference. This process produced a null distribution against which to measure the likelihood of the participant having produced a relative difference as extreme as that which we observed. The permutation test showed that 20 out of 41 participants produced RT with a relative difference exceeding the 95th percentile of random simulations, indicating that the differences in their responses to Accented and Unaccented cues were unlikely to have arisen by chance (Fig. 11). Moreover, there was an imbalance between the two Metre conditions. 17 of the 24 participants in the 4/4 m condition met this threshold, but only 3 of the 17 participants in the 3/4 m condition did. In other words, although there were individual differences in response to Accent, the 4/4 m was generally more conducive to eliciting this effect. Within the subset of 20 participants who appeared to respond to Accent, there was a roughly even share between those who made faster (*n* $= 9$), and those who made slower (*n* $= 11$), responses to Accented cues. A full table of individual results for each participant, including FDR-corrected *p* values for the percentiles, is given in Supplementary Materials in Appendix D, Table D14. There was no statistical relationship between individuals’ Accent relative difference and their Rhythm Score, *r*$= 0.05$, CI $[-0.14, 0.28]$, *p* $= 0.74$, suggesting that individual sensitivity to rhythm, according to the specific measure we used here, was not a factor in whether participants differentiated between Accented and Unaccented cues.

#### Correlational analysis of response to accent

To examine whether response to Accented cues during learning was predictive of the later components of the SRTT, we correlated each participant’s mean Accent relative difference with their change in RT in the Metre test blocks, which was calculated by subtracting test RT from late learning RT, and then standardising that difference by each individual’s mean RT. All reported *p*s are FDR-corrected. We found that Accent relative difference was positively correlated with the slowing of RT in the Phase-Shifted Metre Test Block, *r*$= 0.37$, CI [0.07, 0.56], *p* $= 0.03$, but not in the New Metre test blocks, *r*$= 0.19$, CI $[-0.14, 0.46]$, *p* $= 0.23$. In other words, participants who showed a stronger pattern of Accent-specific responses during learning were also more perturbed by the misalignment of Accent and familiar visual sequence, but their performance was not systematically affected by the introduction of an entirely novel auditory Metre. Finally, Accent relative difference also positively correlated with mean slowing in RT in our implicit learning check, the new visual sequence test, wherein participants saw an unfamiliar visual sequence, *r*$= 0.38$, CI [0.19, 0.54], *p* $= 0.03$. This may indicate that response to Accent could also be associated with sequential learning; however, participants continued to hear their familiar auditory Metre throughout the new visual sequence test, so we cannot disentangle any slowing due to the mismatch between learned auditory cues and unfamiliar visual sequence, as opposed to a change in the latter alone.

#### Variability in accented and unaccented responses

**Fig. 12:**
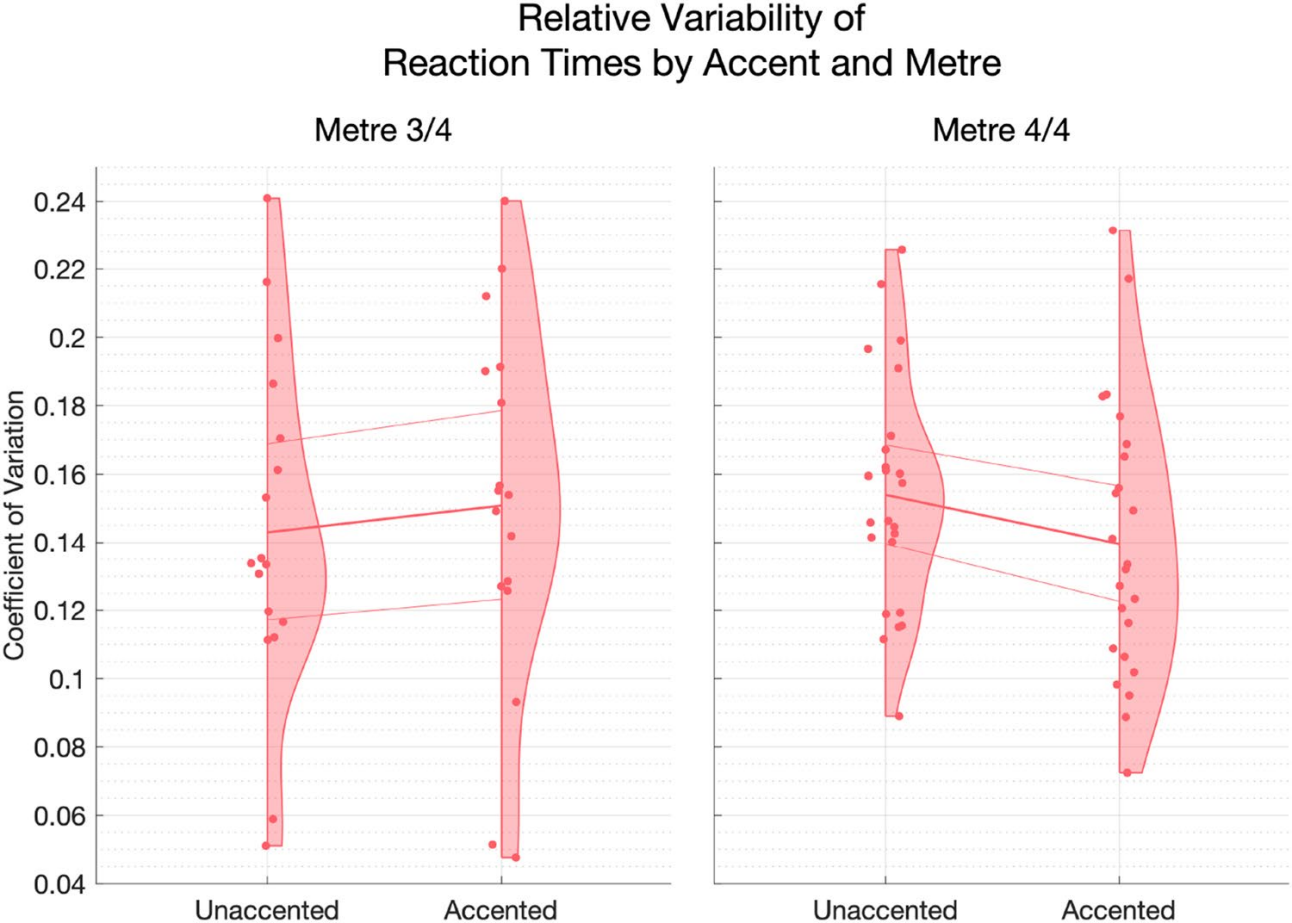
Coefficient of Variation (CV) of responses during learning, calculated for each participant by dividing the standard deviation by mean reaction time, calculated by Accent condition, within each Block, by Participant. Group means are shown by the heavier line, with the thinner lines representing 95% confidence intervals of the mean

Previous work investigating sensorimotor synchronisation to auditory Metre has suggested that actions performed during Accented cues are more temporally stable in comparison to Unaccented cues (Madison, 2014). Given that, in the current experiment, some participants produced systematically faster and others slower RT in response to Accented cues, we wondered whether Accent was associated with reduced *variability*, regardless of the specific direction of Accent-related differences. To this end, we modelled the standard deviation of RT, calculated within Accented and Unaccented responses, within learning Block (Fig. 12). According to residuals diagnostics, the best approach was to model SD as the log of SD, using a linear mixed effect model. Mean RT was included in the model as a control covariate. The responses from Block 1 for a single Participant produced large residual outliers and were removed. We plot the coefficient of variation (CV; SD divided by the Mean), rather than SD, for visual simplicity in Fig. 12. We found, as would be expected, a significant main effect of Mean Reaction Time on log-transformed SD Reaction Time (Estimate = 0.0005, 95% CI [0.0003,0.0007], *t*(1,500.33)= 4.60, *p* $< 0.001$, *R*$^2_\mathrm{sp}$ $= 0.07$). Block, Accent, Metre, and Rhythm Score all failed to meet statistical significance as fixed effects (all *p* $> 0.14$), but there was a significant interaction between Accent and Metre (Estimate = – 0.07, 95% CI [– 0.10,– 0.04], *t*(1,609.11)= -4.08, *p* $< 0.001$, *R*$^2_\mathrm{sp}$ $= 0.01$). Post hoc analysis showed that, although the 3/4 m contrast between Accented and Unaccented responses was non-significant (Estimate = – 1.66, SE = 1.70, *p* $= 0.34$), Accented RT were associated with reduced variability (Mean = 57.95, 95% CI [51.28,64.61], SD = 15.79) in comparison to Unaccented RT (Mean = 65.12, 95% CI [57.82,72.42], SD = 17.29) for participants in the 4/4 m (Estimate = 7.22, SE = 1.55, *p* $< 0.001$). Model details are given in Supplementary Materials in Appendix D, Tables D15 and D16. No other interaction terms improved model fit according to AIC (range – 842.87 to – 789.22).

## Discussion

The current study was motivated to understand how visual sequential learning may be influenced by exposure to two distinct, task-irrelevant auditory metres. Metres can be characterised as a pattern of accented and Unaccented elements or events, and previous behavioural and neural research suggests that these recurring structures dynamically tune attention, such that attentional peaks coincide with accents in time. We hypothesised that RT during visual sequential learning would vary according to accent, and that participants who demonstrated a heightened sensitivity to rhythm would also be more likely to reflect the metrical structure in their SRTT task performance, despite not having received any instruction to explicitly respond to or emulate the auditory rhythm. We devised two metre-specific test blocks that targeted the relationship between the auditory metre heard during training and the visual sequence, speculating that these breakdowns between cross-modal aspects of the learned task would lead to a disruption in RT, as would be expected following a change to the visual sequence itself.

### Effect of metre

We expected to see faster responses according to auditory metric accent status. Instead, we found that response on the basis of accent was almost wholly concentrated in the 4/4 m, and the 3/4 m may have served as something of a control condition. For example, on the group level, we found that participants in the 4/4 m responded more quickly and less variably to accented than unaccented-visual cues. This difference was most pronounced during early learning, and unaccented responses had essentially “caught up” by later blocks. When we examined the magnitude, rather than absolute direction, of variation in response to accent, most participants in the 4/4 m revealed systematic differentiation between accented and unaccented responses. In contrast, just a few participants in the 3/4 m appeared to distinguish between accented and unaccented cues, and their responses to accented cues were associated with equal variability in comparison to unaccented cues.

We suggest that this discrepancy between metre conditions is a strength, rather than a limitation, of the results, because if the accent effect was driven by some other factor, such as cross-modal associative learning (Hoffmann et al., 2001; Kim et al., 2008; Seitz et al., 2007), we should not expect to see any between-metre differences. In fact, in comparison to to 4/4 m, 3/4 m have been shown to produce noisier or inferior performance in behavioural tasks (Abecasis et al., 2005; Bergeson & Trehub, 2006; Drake, 1993), longer latencies in EEG response to metric violations (Abecasis et al., 2005), and a reduced response to accent in magnetoencephalography (Fujioka et al., 2010). In an auditory SRTT with varying ISI, Brandon et al. (2012) found that participants were less able to benefit from beat-based structure during sequential learning in a 3/4 metric condition. Hence, it is possible that 3/4 m-driven effects were unlikely to arise at all in the current study, although our finding here should be replicated, and extended with other types of metres (e.g., groupings of 5 or 7 beats), or within a cross-cultural context: 3/4 m occur less frequently relative to 4/4 ms in popular Western music, where they are primarily found in lullabies and traditional European dance music (e.g., waltzes).

### Accent groupings and possibility of chunking

In addition to metre condition, individual differences also appeared to play a role in how accent emerged as patterning in RT. We had predicted that, due to fluctuations in attending associated with the auditory metre, participants would respond more quickly to visual cues that coincide with auditory accent. This may have been the case for participants in the 4/4 m, especially early on during learning. However, whereas some participants systematically respond more quickly to accented cues throughout the task, we found with permutation tests that others made systematically *slower*, rather than faster, responses to metrically accented-visual cues. Taken at face value, this result contradicts our expectation that accented cues would be associated with increased attention, thereby speeding RT to those cues. One alternative explanation regarding our findings may be that some participants responded to accented cues by pressing for longer or more forcefully, rather than earlier. Our apparatus unfortunately did not record the length or force of key presses, and so we could not investigate possible strategy differences across individual participants. However, it could also be possible that the influence of metre was borne out in these participants’ *chunking* structures, rather than directly in their RT.

Chunks can be described as item or event sets stored “within the same memory code”, which can then be concatenated or reconfigured, so that a long or changing sequence is more efficiently learned and executed as chunks, rather than if it were represented as a series of independent elements (Johnson, 1970; Sternberg et al., 1990). Chunks emerge spontaneously through task practice (Sakai et al., 2004; Shea et al., 2006), but chunking can also be experimentally induced. Verwey and Dronkert (Verwey & Dronkert, 1996), for instance, imposed a chunked pattern in a 9-unit SRTT, by inserting a 750 ms waiting period before presenting the first visual cue of an intended chunk. The authors found that chunk-initial RT were slower than within-chunk RT, similarly to the pattern of results seen for some participants in the current study. Indeed, patterning of relatively slow chunk-initial movements followed by faster within-chunk movements is proposed to be a hallmark of motor sequential learning (Sakai et al., 2004). Du and Clark (2017) suggest that response to stimulus interval timing may have allowed participants in previous SRTT studies to adopt their own temporal grouping strategy, in turn leading to chunked patterns in RT. As we used a fixed inter-stimulus interval, that does not appear to be case here. In a temporal structure SRTT that also had inter-stimulus timing, Tillmann et al. (2011) similarly report the possibility of chunking benefits.

In any case, the extent to which participants made an accent-based distinction during learning was correlated with performance in the subsequent phase-shifted metre test. We also found evidence that participants who distinguished between accented and unaccented cues were disrupted to a greater extent when shown a new visual sequence; however, given our paradigm, it is not possible to dissociate the cross-modal components in the task, and so, we cannot establish with certainty whether this disruption was due to enhanced visual sequential learning, or rather the breakdown between learned correspondences between auditory and visual cues (Mitchel & Weiss, 2011). As similar processes in other domains have been demonstrated, such as the influence of prosody on speech segmentation (Francois & Schön, 2011), sensitivity to metric structure specifically should be studied further as an individual difference of potential import to sequential learning (Anderson et al., 2021).

### Metre tests

Phase-shifting the metre resulted in slower RT, indicating that participants integrated the auditory metre and visual sequence despite no special instruction to do so. There was also a significant interaction with Rhythm Score, which suggested that higher levels of rhythm sensitivity resulted in a greater slowing in RT. We moreover found a correlation between the extent to which individuals differentiated their RT on the basis of accent during learning, and their reduction in RT in response to the phase-shifted metre. Taken together, we should expect to see greater differences as a result of the Phase-Shifted Metre test for participants in the 4/4 m condition, given that is where we found metre-related effects during learning. When modelling on the block level, we find the magnitude of the effect of the test was actually greater in the 3/4 m condition. Descriptive analysis (A)(Supplementary Material, Appendix A) comparing the last 24 RT of Block 8 to the first 24 RT of Block 9 (the test block), however, suggests that participants in the 4/4 m initially slowed more consistently (Mean = +24.45, 95% CI [2.41,46.50]) than did those in the 3/4 m (Mean = +14.51, 95% CI [– 13.67,42.69]), before quickly regaining in speed. In any case, the *lack* of a null effect for the 3/4 m, in light of its inconsistency for accent during learning, could be interpreted as possible evidence that phase-shifting the metre may have had more to do with associative or cross-modal learning than with metre per se. Mitchel & Weiss (2011) explored this topic using a sequential learning paradigm wherein triplets of auditory (tones) and visual (abstract shapes) stimuli co-occurred in an ongoing stream. They found that both types of stimuli could be learned concurrently, whether in fixed association or paired randomly, as long as the boundaries of the triplets were maintained across modes, but mis-aligning the triplets significantly reduced performance (Mitchel & Weiss, 2011). In the current study, if associative learning is indeed what drove the negative effect of phase-shifting the metre, it would not preclude an earlier effect of metric modulation of attention. Specifically, it is possible that at least some sensitivity to rhythm is necessary to perform the cross-modal binding in the first place. Indeed, the Phase-Shifted Metre test block appeared to be very subtle, in that participants who were verbally debriefed following data collection reported no awareness that the correspondence between the sounds and visual cues had changed at that time.

In comparison, the second metre test block, wherein participants heard a completely different metre, was very obvious to participants. The effect of the New Metre test on RT was noisy and prone to inter-individual variability. Looking at RT at the onset of the New Metre test (Fig. 7), we can see that the change from hearing a 3/4–4/4 m (i.e., the New Metre test for those in the 3/4 condition) may have been briefly invigorating for participants with a poorer sense of rhythm, and detrimental for those with a better sense of rhythm. In the former case, the low Rhythm Score participants’ sudden improvements may be related to the otherwise seemingly unchanging nature of the task, although we took no formal reports of boredom and can only speculate. For the higher Rhythm Score participants, on the other hand, it is possible that the unfamiliar 4/4 m was taxing, especially if it is in fact better able to drive entrainment-related effects (i.e., differences in RT on the basis of accent). Participants in the 4/4 m condition, who would start to hear a 3/4 m in this block, generally sped up in RT, so perhaps it was easier for them to “tune out” the less influential 3/4 m.

### Individual sensitivity to rhythm

Participants who scored higher in the perceptual Rhythm Discrimination Task made faster responses in the SRTT, a trend that held in the subset of non-musicians. We had expected that participants with greater sensitivity to rhythm would be more likely to show metre-specific patterns in their RT, as musicians have been previously shown to have enhanced responses in auditory statistical learning (Lerousseau & Schön, 2021). Instead, we found no interaction between block and rhythm score during learning, suggesting that participants tended to improve their responses at similar rates, although this interpretation is complicated by the possibility that high rhythm scoring participants may have been limited by a ceiling effect. Similarly, in our exploratory analysis, we saw no relationship between rhythm score and how much the participant’s responses differed according to accent. We did see interactions between Rhythm Score and the metre tests, and we also found that participants with higher rhythm scores did a little better in the Explicit Recognition Task, though this difference was marginal at best (i.e., 54% rather than 45% correct on average) and was only measured in a subset of participants. In sum, although we found that sensitivity to rhythm is associated with faster RT regardless of musician status, we found mixed evidence for enhanced sequential learning, and no suggestion of a stronger metre-related response in terms of accent.

One possibility is that participants made use of alternative strategies that did not involve their sense of rhythm. For example, some participants may have essentially “ignored”, if non-consciously so, the metric auditory patterns in favour of attending to spatial information or motor feedback. It could also be that the nature of the task did not readily encourage participants to draw on their rhythmic skills. Schultz et al. (2013), for instance, suggest that the benefits of metre may be more pronounced in the encoding and retrieval of memory under explicit conditions. An every-day example of this is the rhythmically stereotyped way in which one recites a long number to memorise it. A future experiment could instruct participants to try to memorise a repeating sequence as quickly as possible, but with only some subjects being advised to attend to the auditory metre, rather than another source, or no aid at all. It is possible that individual differences in sensitivity to rhythm would have a clearer effect under such experimental conditions.

### Limitations

The addition of task-irrelevant tones is reported to improve SRTT performance in musicians (Hoffmann et al., 2001), but others have claimed that only overall RT speed is increased, not visual sequential learning (Conde et al., 2012). As our experiment did not contain a silent condition and we cannot dissociate cross-modal influences, how the presence of task-irrelevant auditory stimuli may have modified visual sequential learning in the current study is uncertain. In a recent study, however, Lagarrigue et al. (2021) employed a fixed response to stimulus interval SRTT to explore this issue. Participants were randomly allocated to either a visual-only or one of several audiovisual conditions of the task. The authors found evidence for enhanced sequential learning in the participants who heard a task-irrelevant sine wave tone during the SRTT, but only if the tone was either concurrently presented with visual cues, or was regularly repeating to form an isochronous rhythm independently of the task. In contrast, the co-presentation of temporally *irregular* auditory stimuli actually appeared to have interfered with visual sequential learning (Lagarrigue et al., 2021). Whether the participants adapted their motor responses to be more rhythmic in the congruent and/or isochronous audiovisual conditions is not reported by Lagarrigue et al. (2021). In any case, although the current work employed a fixed ISI rather than RSI, it is possible that combining the presentation of the visual sequence with auditory rhythms may have promoted, rather than inhibited, sequential learning. This leads to the question concerning individual differences in rhythm perception. Given that we found evidence for faster and potentially more consistent RT, but not a steeper reduction in RT throughout the learning blocks, we can say that sensitivity to rhythm (as measured in the current experiment) is associated with enhanced motor performance, but not necessarily visual sequential learning per se, especially as we did not see a strong relationship between Rhythm Score and slowing of RT following exposure to the New Visual Sequence. Perhaps, other measures of sensitivity to rhythm, especially metric structure, would help to elucidate these results, given that we did find effects of accent, and there are likely to be multiple factors underlying rhythm processing in general (Tierney & Kraus, 2015).

Another limitation to the current study is that we do not experimentally decouple ordinal and temporal patterns. For instance, we could have included a condition where the absolute ordinal series of drum sounds is the same as in the current experiment, except that the ISI are jittered and not regularly timed, leading to temporally unpredictable auditory stimuli that nonetheless express metre-like patterns. Bouwer et al. (2016) found that these irregularly timed, but ordinally structured rhythms can still lead to learning and the violation of auditory expectation, although the authors also report that these effects are more pronounced when the rhythm, and not just the pattern of events, is predictable. Hence, future work should compare between temporally regular and randomly jittered ISI to ascertain whether both forms of auditory pattern exert a similar effect on visual sequential learning. If this were true, it is possible that the results of the metre test blocks would be unchanged across regular and jittered conditions. Conversely, if our current results could be explained by ordinality alone, there is no clear reason why we only found accent-related differences in one of the two metre conditions.

An issue arises in interpreting the diverging results of the metre test blocks, in that we did not vary the block order, and therefore cannot establish that the new metre test was unaffected by the phase-shifted metre test, which always came first. As stated previously, this was a deliberate choice: whereas phase-shifting the auditory metre and visual sequence went unnoticed by participants, changing the metre itself was very obvious. Given the rebound in RT observed in the intermediary learning block between the test blocks, wherein the familiar relationship between the auditory metre and visual sequence was restored, we argue that block order was unlikely to have played a large role in dampening the effect of the second test block, but cannot exclude the possibility that some form of priming might have occurred.

Finally, each metre condition was associated with its own metre-specific background rhythm subdivision sounds. As explained in the Methods section, this was included to make the auditory stimuli more musical and to exaggerate the differences between the metres. Although this subdivision, like the accent patterns, had no currency in terms of task instructions, we cannot disentangle the two metric levels (subdivision and accent) in assessing the overall influence of metre in the current experiment.

## Conclusion

In closing, we have shown that auditory metre can affect visual SRTT task performance, even when inter-stimulus intervals are held constant, as is typical for the standard SRTT. Although we explicitly induced a sense of metre using auditory stimuli, a consideration for any laboratory task is that rhythmic responses are planned and executed differently than non-rhythmic actions (Hogan & Sternad, 2007), and that the human propensity to interpret or subjectively impose metric structure in rhythmic contexts is also well established (B$\mathring{\mathrm{a}}\mathring{\mathrm{a}}$th, 2015; Iversen et al., 2009; ten Hoopen et al., 2008; Vlek et al., 2011), and even apparent in EEG (Brochard et al., 2003; Potter et al., 2009). It is therefore theoretically possible for metre to present as a latent factor in any motor task involving rhythmic timing, even in the absence of overt or external metrical cues. Given the widespread use of the SRTT across many basic and clinical fields, the specific relationship between sequential learning and metre should be investigated more comprehensively, paving the way for cross-disciplinary insights.

## Supplementary Information

Below is the link to the electronic supplementary material.

Supplementary file 1 (pdf 1492 KB)

## Data Availability

Data from the Serial Reaction Time Task and a video of the task are available at https://osf.io/96hrm/ Any other data generated during the current study are available from the corresponding author on reasonable request. The Rhythm Discrimination Task can be viewed online at https://app.gorilla.sc/openmaterials/375726.

## Declarations

### Code availability

Custom code is available from the corresponding author on reasonable request.
