# Supplementary material for: Task-irrelevant auditory metre shapes visuomotor sequential learning
Source: Psychol Res. 2022 Jun 12;87(3):872–93. doi: 10.1007/s00426-022-01690-y (PMC10017598; doi:10.1007/s00426-022-01690-y)
Supplement: Supplementary file 1 — (pdf 1492 KB) [file 426_2022_1690_MOESM1_ESM.pdf]

Springer Nature 2021 LaTeX template

Appendix A Supplementary Material: Analysis of Changes Across and Within Block Boundaries

Previous work investigating sequence learning has found that responses can systematically differ within or across block boundaries. For example, patients may show reduced performance at the beginning of blocks, but can have "caught up" to healthy controls by the end of blocks (Gamble et al., 2014; Nemeth et al., 2013). Hence, looking at localised changes in RT with respect to block boundaries illuminates temporal dynamics that may not be reflected when data are pooled.

We therefore undertook an exploratory descriptive analysis of RT, examining both between- and within-block boundaries to understand how changes in RT may differ across, versus within, block boundaries. This was achieved by calculating the mean difference between the subset of RT at the end (i.e., the last 24 RT = 2 × cycles of the visual sequence) and at the beginning (i.e., the first 24 RT = 2 × cycles of the visual sequence) of each block. We divided participants by median-split Rhythm Score group.

A.1 Learning

Looking at this change in RT *across* the break separating blocks, the High Rhythm group show a tendency to begin a new block slower than their RT were at the end of the previous block (Mean = +10.74, 95% CI [-2.48,23.97], SD = 27.44). The pattern across block boundaries is more variable for the Low Rhythm group (Mean = -4.20, 95% CI [-14.51,6.12], SD = 23.26). In the case of change of RT *within* blocks (i.e., RT at the end of each block compared to its beginning), a stronger pattern of improvement appears for the High Rhythm group, where their mean difference between earlier and later RT within block is consistently negative (Mean = -17.07, 95% CI [-31.74,-2.40], SD = 30.44). For the Low Rhythm group (Mean = +0.19, 95% CI [-9.98,10.36], SD = 22.94), however, the mean difference (within Participant) clusters around 0, indicating that some participants tend towards improvement within learning blocks, whereas others may actually slow in RT as the block progresses.

A.2 Phase-Shifted Metre Test Block

Looking closely at the boundary between Learning and the Phase-Shifted Metre Test, we also examined the differences between subsets of RT at the end of Block 8 and at the beginning of Block 9. There was a substantial overall increase in RT for participants in the High Rhythm group (Mean = +41.16, 95% CI [14.34,67.99], SD = 55.66) but less so for the Low Rhythm group (Mean = +2.34, 95% CI [-17.16,21.83], SD = 43.97). In terms of Metre condition, it seems that participants in the 4/4 Metre (Mean = +24.45, 95% CI [2.41,46.50], SD = 52.20) may have responded more consistently negatively in comparison to the 3/4 Metre (Mean = +14.51, 95% CI [-13.67,42.69], SD =

54.81). Comparing RT *within* Block 9, those in the High Rhythm group generally increased speed within the test block (Mean = -15.06, 95% CI [-33.44,3.32], SD = 38.14), but this comparison was again more variable for participants with lower Rhythm Scores (Mean = +0.33, 95% CI [-25.30,25.97], SD = 57.82), who did not show a within-block improvement on average. Hence, although High Rhythm participants were proportionally more affected by the Phase-Shifted Metre Test, it does not appear that higher sensitivity to Rhythm was an impediment for long beyond the initial change between the visual sequence and auditory metre, and High Rhythm participants may have been able to adapt and regain some speed. This localised pattern of results may help to explain the reduced effect, when viewed at the level of Block, of the test and of Rhythm Score for participants in the 4/4 Metre, given they appear to have first slowed, then quickly rebounded, within a few cycles of the sequence.

A.3 New Metre Test Block

For RT near the boundaries of Blocks 10 and 11, responses to the New Metre test are variable, both for those with lower Rhythm Scores (Mean = -14.92, 95% CI [-51.97,22.14], SD = 83.57), and those with higher Rhythm Scores (Mean = +4.68, 95% CI [-54.67,64.03], SD = 123.14). In the planned modelling on the level of Block, there had been a statistically significant interaction between Block and Metre, suggesting that participants in the 4/4 Metre may have actually sped in RT from Block 10 to 11 ($p < 0.001$). Breaking down the local difference across blocks by Metre group, however, shows that participants in the 3/4 metre (Mean = -1.30, 95% CI [-44.38,41.79], SD = 83.80) and 4/4 metre (Mean = -9.05, 95% CI [-58.12,40.02], SD = 116.20) both produced a mix of faster and slower RT immediately following the onset of Block 11.

We had also modelled a three-way interaction with Block, Metre, and Rhythm Score. Low Rhythm 3/4 participants tended towards faster RT after the onset of the unfamiliar 4/4 metre (Mean = -49.59, 95% CI [-106.43,7.25], SD = 67.99), and High Rhythm 3/4 participants biased instead towards slower RT (Mean = 41.63, 95% CI [-15.87,99.13], SD = 74.80). This contrast does not appear to occur between Low Rhythm (Mean = 4.89, 95% CI [-45.55,55.33], SD = 87.36) and High Rhythm (Mean = -28.57, 95% CI [-136.49,79.35], SD = 150.86) participants in the 4/4 Metre. The confidence intervals for all four groups accommodate 0, affirming the overall mixed result of the New Metre test block.

As participants continued to hear the New Metre test, those with lower Rhythm Scores in the 3/4 Metre tended to slow down from the beginning to the end of Block 11 (Mean = +51.13, 95% CI [-2.53,104.80], SD = 64.19). Participants with higher Rhythm Scores in the 3/4 Metre showed a stronger pattern of improvement (Mean = -37.41, 95% CI [-90.98,16.15], SD = 69.69). For the 4/4 Metre, however, both Low Rhythm (-11.00, 95% CI [-46.58,24.57], SD = 61.62) and High Rhythm (Mean = -5.62, 95% CI [-58.06,46.81], SD = 73.29) participants varied considerably in their responses throughout the New Metre Test block.

Springer Nature 2021 LaTeX template

A.4 Discussion

During Learning, participants in the median-split higher Rhythm Score group tended to slow at the beginning of new blocks. These High Rhythm responses show a staircase-like pattern with a steady, decreasing trend in RT, as can be seen in Figure A1. By contrast, the responses of participants in the Low Rhythm group followed a flatter and much more variable trajectory throughout the SRTT. We cannot be sure whether the staircase pattern is due to sensitivity to rhythm per se. For example, it could be that people with higher rhythm sensitivity also have more experience with playing music or computer gaming, leading to an enhanced stability of motor responses for prolonged periods (i.e., an entire block of 120 responses). This does not in itself explain why the High Rhythm group begin each Learning block slower than the previous block ended, but it is possible that such a pattern could reflect a within-block ceiling effect in performance. These descriptive insights indicate possible between-group differences in behaviour across block boundaries that, whether due to differences in motor performance or rhythm sensitivity specifically, should be formally confirmed in a new data set.

In the analysis of between-block boundaries between Block 8 and the Phase-Shifted Metre test, it appears that the true effect on RT is actually dampened when modelling full blocks, perhaps because participants rapidly adjust to the new correspondence between the metre and visual sequence after a brief, initial disruption. On the other hand, we also observed the aforementioned staircase pattern in High Rhythm participants, with slowing at the beginning of almost every new block, which throws the effect of the test block into question. We note, however, the slowing of RT in transition to Phase-Shifted Metre for High Rhythm participants (Mean = 41.16, 95% CI [14.34,67.99]) is much greater in magnitude in comparison to during Learning blocks (Mean = 10.74, 95% CI [-2.48,23.97]). Moreover, the reinstatement of the familiar, learned pairing between visual sequence and auditory metre in Block 10 is the only block where High Rhythm participants show a slight trend towards faster between-block differences in RT. Finally, with regards to the New Metre Test, neither breaking down performance by group, nor by responses at the beginnings and ends of blocks 10 and 11, revealed any further insights. We therefore have higher confidence that the null effect of the New Metre Test observed in the planned contrasts was not the result of pooling by Block.

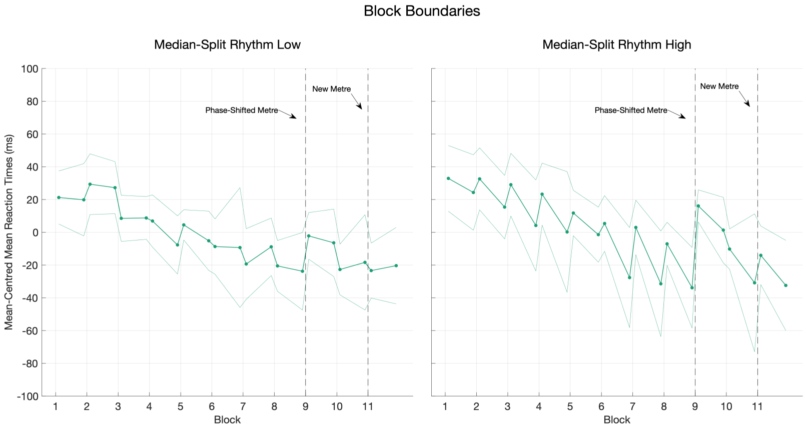

Fig. A1: Mean-centred mean reaction times (calculated within participant) across the complete experiment, sampled as only the first and last two cycles (n = 24 reaction times) for each block. Responses are summarised by median-split Rhythm Group. Group means are shown by dots, with smaller lines representing 95% confidence intervals of the mean.

Appendix B Goldsmiths Musical Sophistication Index

Table B1: Spearman's Rho Correlations between selected sub-scales and General Sophistication score of the Goldsmiths Musical Sophistication Index and Rhythm Score, with data available for 16 participants.

Goldsmiths Musical Sophistication Index and Rhythm Score					
			Confidence Intervals		
		r_s	2.5%	97.5%	p
Rhythm Score	Perceptual Abilities	0.75	0.22	0.91	< 0.001
	Musical Training	0.68	0.32	0.87	0.004
	General Sophistication	0.64	0.1	0.87	0.007

Appendix C Planned Analysis

C.1 Linear Mixed Modelling Details

C.1.1 Effect of Accent During Learning

Table C2: Details for the linear mixed model of reaction times during the Learning Blocks.

Linear Mixed Model fit by Restricted Maximum Likelihood Estimation. Model formula: Reaction Times ~ 1 + Block + Accent + Metre + Rhythm Score + Accent:Metre:Block + (1 + Block |Participant)						
Observations:	36104					
Dependent Variable:	Reaction Times (ms)					
Model Fit	AIC:	422939.10				
	BIC:	423049.53				
Pseudo-R^2	Fixed Effects:	0.10				
	Total:	0.58				
Fixed Effects						
		Confidence Intervals				
	Est.	2.50%	97.50%	t	DF	p
(Intercept)	493.56	446.28	540.84	20.46	39.12	< 0.001
Block	-8.28	-12.42	-4.13	-3.91	42.42	< 0.001
Accent	-4.50	-10.92	1.92	-1.37	36019.18	0.17
Metre	-62.13	-124.31	0.04	-1.96	39.37	0.06
Rhythm Score	-38.28	-65.95	-10.61	-2.71	37.99	0.01
Accent × Metre	21.15	12.49	29.80	4.79	36018.83	< 0.001
Block × Accent	0.41	-0.85	1.68	0.64	36018.99	0.52
Block × Metre	5.56	0.12	11.00	2.00	43.07	0.05
Block × Accent × Metre	-2.65	-4.36	-0.95	-3.05	36018.70	0.002
p-Values are calculated using Satterthwaite degrees of freedom.						
Random Effects						
Group	Parameter	SD				
Participant (41)	(Intercept)	98.12				
	Block	8.45				
Residual		84.14				
Intraclass Correlation Coefficient:	0.58					

Table C3

Contrasts of Estimated Marginal Means for Accent					
Contrast: Accented - Unaccented	Estimate	SE	DF	t	p
3/4 Metre	2.63	1.47	36018	1.79	0.07
4/4 Metre	-6.52	1.34	36018	-4.88	< 0.001

Trend Analysis of Marginal Means for Block					
	Trend	SE	DF	t	p
3/4 Metre Accented	-8.28	2.12	42.4	-3.91	0.001
3/4 Metre Unaccented	-7.86	2.08	39.8	-3.78	0.002
4/4 Metre Accented	-2.72	1.80	44.0	-1.51	0.55
4/4 Metre Unaccented	-4.96	1.75	39.4	-2.84	0.03

p-Values are calculated using Satterthwaite degrees of freedom and corrected using the Bonferroni method.

C.1.2 Phase-Shifted Metre Test

Table C4: Details for the linear mixed model of reaction times contrasting Block 8 and Block 9 (Phase-Shifted Metre Test).

Linear Mixed Model fit by Restricted Maximum Likelihood Estimation. Model formula: Reaction Times ~ 1 + Rhythm Score + Metre + Block + Rhythm Score:Metre:Block + (1 |Participant)

Observations:	9123					
Dependent Variable:	Reaction Times (ms)					
Model Fit	AIC:	106590.55				
	BIC:	106661.74				
Pseudo-R^2	Fixed Effects:	0.20				
	Total:	0.60				
Fixed Effects						
		Confidence Intervals				
	Est.	2.5%	97.5%	t	DF	p
(Intercept)	440.30	395.10	485.49	19.09	37.28	< 0.001
Rhythm Score	-73.93	-127.82	-20.03	-2.69	37.29	0.01
Metre	-30.54	-89.14	28.06	-1.02	37.27	0.31
Block	6.47	1.02	11.93	2.33	9078.05	0.02
Rhythm Score × Metre	45.90	-17.76	109.57	1.41	37.28	0.17
Block × Metre	-2.63	-9.68	4.41	-0.73	9078.06	0.46
Rhythm Score × Metre × Block 8	8.71	2.15	15.27	2.60	9078.06	0.009
Rhythm Score × Metre × Block 9	6.06	2.02	10.10	2.94	9078.01	0.003
p-Values are calculated using Satterthwaite degrees of freedom.						
Random Effects						
Group	Parameter	SD				
Participant (41)	(Intercept)	91.98				
Residual		82.45				
Intraclass Correlation Coefficient:	0.55					

Table C5

Contrasts of Estimated Marginal Means for Block					
Contrast: Block 8 - Block 9	Estimate	SE	DF	t	p
3/4 Metre	-6.47	2.78	9078	-2.33	0.02
4/4 Metre	-3.84	2.27	9078	-1.69	0.09
Trend Analysis of Marginal Means for Rhythm Score					
	Trend	SE	DF	t	p
3/4 Metre Block 8	-5.20	1.93	37.3	-2.69	0.04
3/4 Metre Block 9	-4.58	1.93	37.3	-2.37	0.09
4/4 Metre Block 8	-1.97	1.22	37.3	-1.62	0.45
4/4 Metre Block 9	-1.54	1.22	37.3	-1.27	0.85

p-Values are calculated using Satterthwaite degrees of freedom and corrected using the Bonferroni method.

C.1.3 New Metre Test

Table C6: Details for the linear mixed model of reaction times contrasting Block 10 and Block 11 (New Metre Test).

Linear Mixed Model fit by Restricted Maximum Likelihood Estimation. Model formula: Reaction Times ∼ 1 + Rhythm Score + Metre + Block + Rhythm Score:Block:Metre + (1 |Participant)

Observations:	8986					
Dependent Variable:	Reaction Times (ms)					
Model Fit	AIC:	106109.78				
	BIC:	106180.81				
Pseudo-R^2	Fixed Effects:	0.13				
	Total:	0.58				

Fixed Effects

		Confidence Intervals				
	Est.	2.5%	97.5%	t	DF	p
(Intercept)	438.41	393.01	483.81	18.93	37.29	< 0.001
Rhythm Score	-90.59	-144.64	-36.54	-3.28	37.32	0.002
Metre	-31.51	-90.27	27.25	-1.05	37.29	0.30
Block	1.41	-4.40	7.22	0.48	8941.01	0.63
Rhythm Score × Block	11.47	4.43	18.51	3.19	8941.02	0.001
Block × Metre	-12.33	-19.86	-4.80	-3.21	8941.02	0.001
Rhythm Score × Metre × Block 10	71.09	7.24	134.95	2.18	37.31	0.04
Rhythm Score × Metre × Block 11	55.88	-7.97	119.73	1.72	37.30	0.09

p-Values are calculated using Satterthwaite degrees of freedom.

Random Effects

Group	Parameter	SD
Participant (41)	(Intercept)	92.07
Residual		87.77
Intraclass Correlation Coefficient:	0.52	

Table C7

Contrasts of Estimated Marginal Means for Block

Contrast: Block 10 - Block 11	Estimate	SE	DF	t	p
3/4 Metre	-1.41	2.96	8941	-0.48	0.63
4/4 Metre	10.92	2.45	8941	4.46	< 0.001

Trend Analysis of Marginal Means for Rhythm Score

	Trend	SE	DF	t	p
3/4 Metre Block 10	-6.36	1.94	37.3	-3.29	0.008
3/4 Metre Block 11	-5.55	1.94	37.3	-2.87	0.03
4/4 Metre Block 10	-1.37	1.22	37.3	-1.12	1.0
4/4 Metre Block 11	-1.63	1.22	37.3	-1.34	0.75

p-Values are calculated using Satterthwaite degrees of freedom and corrected using the Bonferroni method.

C.1.4 Comparison of Block 8 and Block 10

Table C8: Details for the linear mixed model of reaction times in Blocks 8 and 10.

Linear Mixed Model fit by Restricted Maximum Likelihood Estimation. Model formula: Reaction Times ∼ 1 + Rhythm Score + Metre + Block + Rhythm Score:Block:Metre + (1 |Participant)

Observations:	9026					
Dependent Variable:	Reaction Times (ms)					
Model Fit	AIC:	105962.11				
	BIC:	106033.19				
Pseudo-R^2	Fixed Effects:	0.12				
	Total:	0.62				

Fixed Effects

		Confidence Intervals				
	Est.	2.5%	97.5%	t	DF	p
(Intercept)	441.16	393.07	489.24	17.98	37.26	< 0.001
Rhythm Score	-74.42	-131.71	-17.12	-2.55	37.26	0.02
Metre	-31.33	-93.59	30.92	-0.99	37.25	0.33
Block	-2.96	-8.60	2.68	-1.03	8981.07	0.30
Rhythm Score × Block	-15.74	-22.55	-8.93	-4.53	8981.10	< 0.001
Block × Metre	0.83	-6.45	8.12	0.22	8981.10	0.82
Rhythm Score × Metre × Block 8	46.13	-21.55	113.81	1.34	37.25	0.19
Rhythm Score × Metre × Block 10	70.39	2.70	138.08	2.04	37.26	0.05

p-Values are calculated using Satterthwaite degrees of freedom.

Random Effects

Group	Parameter	SD
Participant (41)	(Intercept)	97.64
Residual		84.77
Intraclass Correlation Coefficient:	0.57	

Springer Nature 2021 LaTeX template

Table C9

Contrasts of Estimated Marginal Means for Block					
Contrast: Block 8 - Block 10	Estimate	SE	DF	*t*	*p*
3/4 Metre	2.96	2.88	8981	1.03	0.30
4/4 Metre	2.13	2.35	8981	0.9	0.37
**Contrasts of Estimated Marginal Means for Metre**					
Contrast: 3/4 Metre - 4/4 Metre	Estimate	SE	DF	*t*	*p*
Block 8	31.3	31.8	37.2	0.99	0.33
Block 10	30.5	31.8	37.2	0.96	0.34
**Trend Analysis of Marginal Means for Rhythm Score**					
	Trend	SE	DF	*t*	*p*
3/4 Metre Block 8	-5.22	2.05	37.3	-2.55	0.02
3/4 Metre Block 10	-6.33	2.05	37.3	-3.08	0.004
4/4 Metre Block 8	-1.98	1.29	37.2	-1.54	0.13
4/4 Metre Block 10	-1.39	1.29	37.2	-1.08	0.29

p-Values are calculated using Satterthwaite degrees of freedom and corrected using the Bonferroni method.

C.1.5 Implicit and Explicit Learning Tests

New Visual Sequence Test

Immediately after completing the main task, visual sequential learning was confirmed with an additional SRTT block that combined the learned auditory metre with an unfamiliar series of visual cues, corresponding to the canonical test block in the standard SRTT. RT in this task were compared with those from Blocks 8 and 10 in the main SRTT as a sanity check that the new visual sequence was performed more slowly than the learned visual sequence.

Table C10: Details for the linear mixed model of reaction times during the Late Learning Blocks (Blocks 8 and 10) and the New Visual Sequence Test.

Linear Mixed Model fit by Restricted Maximum Likelihood Estimation.
Model formula: Reaction Times ~ 1 + New Visual Sequence + Metre + Rhythm Score + Rhythm Score:Metre:New Visual Sequence + (1 + New Visual Sequence |Participant)

Observations:	12581					
Dependent Variable:	Reaction Times (ms)					
Model Fit	AIC:	148123.06				
	BIC:	148197.46				
Pseudo-R^2	Fixed Effects:	0.12				
	Total:	0.59				
**Fixed Effects**						
		Confidence Intervals				
	Est.	2.50%	97.50%	t	DF	p
(Intercept)	439.15	394.17	484.14	19.13	37.07	< 0.001
New Visual Sequence	26.58	20.94	32.21	9.24	12539.33	< 0.001
Metre	-30.55	-88.89	27.79	-1.03	37.07	0.08
Rhythm Score	-82.10	-135.78	-28.42	-3.0	37.08	0.005
Rhythm Score × Metre	58.15	-5.27	121.56	1.80	37.08	0.08
Metre × New Visual Sequence	18.12	10.97	25.26	4.97	12538.50	< 0.001
Rhythm Score × New Visual Sequence × 3/4 Metre	21.47	14.86	28.08	6.36	12538.20	< 0.001
Rhythm Score × New Visual Sequence × 4/4 Metre	3.93	-0.11	7.97	1.91	12537.29	0.06
p-Values are calculated using Satterthwaite degrees of freedom.						
**Random Effects**						
Group	Parameter	SD				
Participant (41)	(Intercept)	91.74				
Residual		86.46				
Intraclass Correlation Coefficient:	0.53					

Table C11

Contrasts of Estimated Marginal Means for New Visual Sequence					
Contrast: Late Learning - New Visual Sequence	Estimate	SE	DF	t	p
3/4 Metre	-26.60	2.88	12539	-9.24	< 0.001
4/4 Metre	-44.70	2.24	12537	-19.98	< 0.001

Trend Analysis of Marginal Means for Block					
	Trend	SE	DF	t	p
3/4 Metre Late Learning	-5.77	1.92	37.1	-3.00	0.02
3/4 Metre New Visual Sequence	-4.26	1.93	37.6	-2.21	0.13
4/4 Metre Late Learning	-1.68	1.21	37.1	-1.39	0.69
4/4 Metre New Visual Sequence	-1.41	1.21	37.5	-1.16	1.0

p-Values are calculated using Satterthwaite degrees of freedom and corrected using the Bonferroni method.

Explicit Recognition Test

Next, a secondary task was administered to gauge explicit knowledge of the SRTT learned visual sequence. Note that this task was introduced for only one of the two testing sites, and hence only a subset of participants who performed the main task took part ($n = 26$). The purpose of the task was to ensure that the cross-modal aspects of our modified SRTT did not result in increased explicit awareness, in comparison to the standard SRTT. The explicit recognition task followed a similar format to the SRTT in terms of visual presentation; however, the trials were self-paced, rather than strictly timed, and there was no auditory component. In each trial, the first two or three elements of a visual sequence were shown before the participant was prompted to guess the next element by responding with its key. After guessing, they were asked "Did you know what the next location would be?". There were forty-eight trials in total, of which 25% (12) contained truly familiar segments from the learned visual sequence. Of these, 50% (6) were presented in a grouping that reflected the participant's own SRTT metre condition (i.e., a group of either three or four). Only data from trials containing the truly familiar segments were retained, and the dependent variable was correct/incorrect responses. We modelled likelihood of a Correct Response in the Explicit Recognition Task data as a binomial distribution using the logit link function. Possible fixed effect terms were Metre, length of segment (i.e., 3 or 4 elements), and self-reported familiarity with the segment as factors; and Rhythm Score as a covariate.

Table C12: Details for the generalised linear mixed model of correct answers in the Explicit Recognition Test, fit as a binomial distribution with logit link by maximum likelihood (Adaptive Gauss-Hermitte Quadrature). This model contains observations for participants who were excluded from the Serial Reaction Time Task due to high rates of anticipated correct responses.

Generalised Linear Mixed Model fit as a binomial distribution with logit link by maximum likelihood. Model formula: Correct $\sim$ 1 + Rhythm Score + (1 |Participant)					
Observations:	624				
Dependent Variable:	Answer Correct				
Model Fit	AIC:	865.6			
	BIC:	878.91			
Pseudo-R^2	Fixed Effects:	0.01			
	Total:	0.01			
Fixed Effects					
		Confidence Intervals			
	Est.	2.5%	97.5%	z	p
(Intercept)	0.07	-0.09	0.23	0.88	0.38
Rhythm Score	-0.17	-0.34	-0.01	-2.11	0.03
Random Effects					
Group	Parameter	SD			
Participant (26)	(Intercept)	0.07			
Intraclass Correlation Coefficient:	0				

Table C13: Details for the generalised linear mixed model of correct answers in the Explicit Recognition Test, fit as a binomial distribution with logit link by maximum likelihood (Adaptive Gauss-Hermitte Quadrature). This model contains only observations for participants who were not excluded from the Serial Reaction Time Task due to high rates of anticipated correct responses.

Generalised Linear Mixed Model fit as a binomial distribution with logit link by maximum likelihood. Model formula: Correct $\sim$ 1 + Rhythm Score + (1 |Participant)

Observations:	576				
Dependent Variable:	Answer Correct				
Model Fit	AIC:	800.26			
	BIC:	813.33			
Pseudo-R^2	Fixed Effects:	0.01			
	Total:	0.01			
**Fixed Effects**					
		Confidence Intervals			
	Est.	2.5%	97.5%	z	p
(Intercept)	0.04	-0.13	0.2	0.42	0.67
Rhythm Score	-0.17	-0.34	0	-1.98	0.05
**Random Effects**					
Group	Parameter	SD			
Participant (24)	(Intercept)	0.06			
Intraclass Correlation Coefficient:	0				

Springer Nature 2021 LaTeX template

Appendix D Exploratory Analysis

D.1 Permutation Tests of Accent Relative Difference

Table D14: Summary statistics for the results of the permutation test (n= 10,000) of relative difference between reaction times for Accented and Unaccented auditory cues. For each iteration, relative difference was calculated within response Key, within Block, and within participant to produce a simulated null distribution by which to test the likelihood of obtaining a pattern of responses (i.e., faster or slower reaction times on the basis of accent) as extreme as that observed.

Participant	Metre	Mean Relative Difference (Accented - Unaccented)	Percentile	p	Accented RT
1	4/4	7.30	100	<0.001	Faster
2	4/4	10.58	100	<0.001	Faster
3	4/4	6.31	100	<0.001	Slower
4	4/4	3.82	100	<0.001	Faster
5	3/4	2.95	100	<0.001	Slower
6	4/4	2.28	100	<0.001	Slower
7	4/4	3.50	100	<0.001	Slower
8	4/4	11.00	100	<0.001	Slower
9	4/4	3.09	100	<0.001	Faster
10	4/4	9.10	100	<0.001	Faster
11	4/4	7.10	100	<0.001	Slower
12	4/4	7.76	100	<0.001	Slower
13	3/4	3.70	99.99	0.001	Faster
14	4/4	3.76	99.97	0.001	Faster
15	4/4	3.48	99.97	0.001	Slower
16	4/4	3.07	99.86	0.004	Faster
17	4/4	2.39	99.74	0.007	Slower
18	4/4	2.62	99.68	0.008	Faster
19	3/4	2.01	99.44	0.012	Slower
20	4/4	2.97	96.47	0.073	Slower
21	4/4	1.82	94.35	0.110	Faster
22	3/4	4.69	93.78	0.111	Faster
23	3/4	2.54	93.78	0.111	Slower
24	4/4	3.38	92.04	0.136	Slower
25	3/4	0.89	90.85	0.150	Slower
26	3/4	1.69	86.81	0.208	Slower
27	3/4	1.68	71.42	0.434	Faster
28	4/4	1.94	68.48	0.462	Slower
29	3/4	0.73	64.81	0.498	Faster
30	4/4	2.91	53.58	0.614	Slower
31	3/4	1.55	54.31	0.614	Faster
32	4/4	2.14	42.36	0.739	Slower
33	3/4	1.59	34.64	0.812	Slower
34	4/4	1.55	29.34	0.852	Faster
35	3/4	1.13	15.54	0.989	Slower
36	4/4	0.73	0.04	1.000	Faster
37	3/4	1.45	6.69	1.000	Faster
38	3/4	3.54	0.12	1.000	Slower
39	3/4	0.94	0.57	1.000	Slower
40	3/4	1.09	2.14	1.000	Faster
41	3/4	0.71	4.17	1.000	Slower

RT = Reaction times. p-Values are FDR corrected.

D.2 Linear Mixed Modelling Details

D.2.1 Effect of Accent on Variability During Learning

Table D15: Details for the linear mixed model of log-transformed standard deviation of reaction times during the Learning Blocks.

Linear Mixed Model fit by Restricted Maximum Likelihood Estimation.
Model formula: Log Standard Deviation Reaction Times ~ 1 + Mean Reaction Times + Block + Accent + Metre + Rhythm Score + Accent:Metre + (1 + Block |Participant)

Observations:	654					
Dependent Variable:	Standard Deviation Reaction Times (ms)					
Model Fit	AIC:	-872.23				
	BIC:	-831.88				
Pseudo-R^2	Fixed Effects:	0.12				
	Total:	0.65				
**Fixed Effects**						
		Confidence Intervals				
	Est.	2.50%	97.50%	t	DF	p
(Intercept)	1.52	1.41	1.64	25.20	191.44	< 0.001
Mean Reaction Times	0.0005	0.0003	0.0007	4.60	500.33	< 0.001
Block	0.003	-0.0009	0.01	1.48	627.23	0.14
Accent	0.013	-0.01	0.04	1.01	608.78	0.31
Metre	0.04	-0.05	0.13	0.88	40.72	0.38
Rhythm Score	-0.02	-0.06	0.02	-0.94	39.78	0.35
Accent × Metre	-0.07	-0.10	-0.04	-4.08	609.11	< 0.001
p-Values are calculated using Satterthwaite degrees of freedom.						
**Random Effects**						
Group	Parameter	SD				
Participant (41)	(Intercept)	0.13				
Residual		0.11				
Intraclass Correlation Coefficient:	0.60					

Table D16

Contrasts of Estimated Marginal Means for Accent

Contrast: Accented - Unaccented	Estimate	SE	DF	t	p
3/4 Metre	-1.66	1.70	40	-0.97	0.34
4/4 Metre	7.22	1.55	39.9	4.66	< 0.001

Dependent Variable is back-transformed from log. p-Values are calculated using Satterthwaite degrees of freedom and corrected using the Bonferroni method.

Appendix E Extended Figures

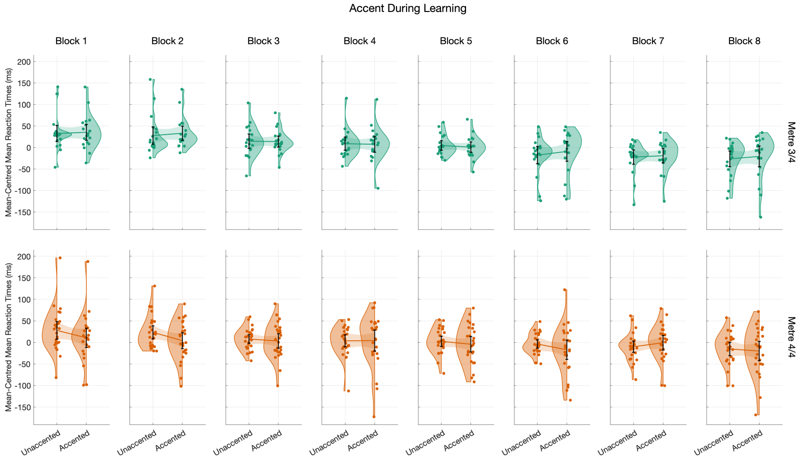

Fig. E2: Mean-centred mean reaction times (calculated within participant) by Accent during Learning. The data are summarised by Metre across rows, and Block across columns. Group means are shown by lines, with the error bars and shaded regions representing 95% confidence intervals of the mean.
